# Docosahexaenoate-enriched fish oil and medium chain triglycerides shape the feline plasma lipidome and synergistically decrease circulating gut microbiome-derived putrefactive postbiotics

**DOI:** 10.1371/journal.pone.0229868

**Published:** 2020-03-12

**Authors:** Matthew I. Jackson, Dennis E. Jewell

**Affiliations:** Pet Nutrition Center, Hill’s Pet Nutrition, Inc., Topeka, Kansas, United States of America; Universite du Quebec a Montreal, CANADA

## Abstract

The purpose of this study was to examine the influence of medium-chain fatty acid-containing triglycerides (MCT), long-chain polyunsaturated fatty acid-containing triglycerides, and their combination on the plasma metabolome of cats (*Felis catus*), including circulating microbiome-derived postbiotics. After a 14-day lead-in on the control food, cats were randomized to one of four foods (control, with 6.9% MCT, with fish oil [FO; 0.14% eicosapentaenoate, 1.0% docosahexaenoate], or with FO+MCT; n = 16 per group) for 28 days. Analysis of plasma metabolites showed that the addition of FO and MCT led to synergistic effects not seen with either alone across a number of lipid classes, including fatty acids, acylcarnitines, and acylated amines including endocannabinoids. Notably, the FO+MCT group had an increase in ketone body production relative to baseline and beyond that seen with MCT alone. N-acyl taurines, the accumulation of which has been implicated in the onset of type 2 diabetes, were significantly decreased in the FO+MCT group. Significant decreases in the gut microbiome-derived postbiotic classes of indoles/indolic sulfates and phenols/phenolic sulfates were observed only the FO+MCT group. Overall, the combination of MCT and FO led to number of changes in plasma metabolites that were not observed with either oil alone, particularly in postbiotics.

## Introduction

Dietary fatty acids esterified in triglyceride form are an important source of nutritional energy and consist of diverse carbon chain lengths and degrees of unsaturation. Found at orthogonal extremes of these characteristics are the medium-chain saturated fatty acids (MCFAs) and the long-chain polyunsaturated fatty acids (LCPUFAs), each of which is typically consumed in triglyceride form (MCT and LCPUT, respectively).

Differences between LCPUT and MCT are apparent from the outset of their catabolism; MCTs are hydrolyzed to MCFAs by pancreatic lipase with an avidity exceeding that for long chain fatty acid-containing triglycerides [1]. Additionally, LCPUFAs are re-esterified into triglyceride form after absorption into intestinal epithelial cells, incorporated into chylomicrons before circulating through the periphery and their subsequently arrival at the liver where they can be incorporated into low density lipoproteins (LDL). In contrast, MCFAs are absorbed directly to the liver via portal circulation. During catabolic energy production, MCFAs do not require carnitine to be shuttled into mitochondria and are extensively converted to ketone bodies in the liver, which are then exported to the systemic circulation, leading to ketonemia and the designation of MCTs as “ketogenic” [2]. Although long-chain saturated fatty acids (LCSFAs) are extensively used as sources of energy in the periphery, they must be initially catabolized in the peroxisome or transported into mitochondria via conjugation with carnitine [3]. During catabolism, LCSFAs generate acetyl-CoA rather than ketone bodies. LCPUFAs are not catabolized to energy to the same degree as LCSFA but rather are preferentially incorporated into phospholipids and subsequently lipid membranes of cells [4]. Consumption of both MCT and LCPUT modulates physiology beyond provision of dietary energy. Whereas MCTs are prominent in nutritional prescriptions for modifying metabolic and overweight status, LCPUTs are often employed to alter the inflammatory state. Both metabolic or overweight status and inflammation are known to interact with the gut microbiome to influence the health of the host.

The ability of the gut microbiome to influence host health and metabolism has become well-established in recent years. Recent studies have established an association between the gut microbiome and blood lipids in humans, mice, and rats [5, 6]. Different food types can alter the host gut microbiome, including types of dietary lipids [7–9]. The gut microbiome has been shown to generate metabolites, also known as postbiotics, that can be absorbed across the colon into the host systemic circulation [10, 11]. Postbiotics are metabolic products of the gut microbiome that originate from microbial catabolism of undigested food that bypassed absorption in the small intestine. For example, microbial fermentation of fiber generates short-chain fatty acid postbiotics that influence host health. Relevant to the current study, gut microbial putrefaction of the amino acids tryptophan and phenylalanine or tyrosine leads to the production of postbiotic indoles and phenols, respectively, and their absorption in the systemic circulation of the host [12]. Often these microbial postbiotics are conjugated to sulfate by the intestinal epithelium or liver during reabsorption. Postbiotics derived from microbial putrefaction of amino acids can have deleterious consequences to host health, particularly as risk factors for inflammation and renal disease [13].

MCTs modify the gut microbiome and this has been proposed as a mode of action for these fats to improve the metabolic health of obese individuals [14]. Additionally, MCTs influence neutrophil functions, including phagocytosis and oxidative bacterial killing, which may impact the makeup of gut microbiota [15]. MCTs have been shown to positively impact gut barrier integrity in an inflammatory model [16, 17], which could influence the appearance of microbial metabolites in systemic circulation [18]. Relevant to intestinal inflammation, MCTs reduce the activity of bacterial-responsive inflammatory pathways and have shown efficacy in decreasing colitis [19]. LCPUFA(n3) such as eicosapentaenoate (EPA; C20:5n3) and docosahexaenoate (DHA; C22:6n3) are notable for their impact on inflammatory processes, and intake of these oils has recently been shown to influence composition of the gut microbiome [8, 20]. Consumption of fish and krill seafood oils rich in polyunsaturated fatty acids has been shown to impact microbiome community structure as well as the abundances of particular microbes that are associated with physiologic traits such as obesity [8, 21] and brain aging [22]. Whereas LCPUFA(n3) are associated with increased beneficial gut microbiota, LCPUFA(n6) have been shown to have detrimental effects on gut barrier function and microbiome composition [7, 23]. Consumption of fish oil (FO), a source of LCPUT(n3), and MCT fats together produces an interaction that modulates physiological processes and disease risk. Combinations of long chain triglycerides and MCT have synergistic effects on intestinal adaptation and morphology [24], MCT may potentiate the benefits of FO on cardiovascular risk factors [25], and MCT modulates the effect of FO on inflammatory processes [26–29].

Felines are nutritionally considered obligate carnivores due in part to their inability to endogenously synthesize certain fatty acids (e.g., arachidonate). Genomic investigations have recently characterized genetic elements associated with domestication of the housecat (*Felis catus*) compared with wildcats and found genes involved in lipid metabolism to be altered, including representatives of the fatty acid beta oxidation, ether lipid metabolism, and peroxisomal lipid metabolism pathways [30]. A few published studies have characterized the feline circulating metabolome [31–33], however, without specific focus on lipids. The levels and types of circulating complex lipid classes are not extensively documented in the cat. Given their interactive effects on physiology and use as dietary fats, it is of interest to characterize the co-metabolism of dietary FO and MCT. In this study, we documented the impact on the feline housecat plasma lipidome caused by consumption of dietary triglycerides containing fatty acids from opposite ends of the spectra of chain length and degree of unsaturation. MCT and n-3 LCPUT were fed alone or in combination with the objective of determining the degree to which MCT and LCPUT impacted circulating complex lipids in cats as part of a complete maintenance food balanced for total dietary fat, protein, and carbohydrate. In addition, the effect of these dietary oils on the gut microbiome was analyzed via the changes in circulating putrefactive postbiotics along with their host-conjugated sulfates.

## Materials and methods

### Ethics statement

The study protocol was reviewed and approved by the Institutional Animal Care and Use Committee, Hill’s Pet Nutrition, Topeka, KS, USA (Protocol Number: FP579.1.3.0-A-F-D-ADH) and complied with the National Institutes of Health guide for the care and use of laboratory animals as well as the guides for the care and use of laboratory animals from the US National Research Council and US Public Health Service [34]. Inclusion criteria was for healthy cats, defined as no evidence of chronic systemic disease from physical examination, complete blood count, serum biochemical analyses, urinalysis, or fecal examination for parasites. No invasive procedures or procedures involving suffering were utilized in this study. Sedation was used prior to phlebotomy in order to minimize stress on cats.

### Food formulation and production

Four dry extruded foods, which met the feline maintenance nutrition requirements outlined by the Association of American Feed Control Officials and National Research Council, were tested, all with the same base formula consisting primarily of wet chicken meat, corn gluten meal, whole wheat, and pork fat. Pork fat in the control (CON) food was replaced with MCT, FO, or MCT and FO in the test foods used in this study (S1 Table). CAPTEX-355 (ViaChem Inc, Plano, TX, USA) was chosen as the source of MCT as it was enriched for caprylate (C8:0) over caprate (C10:0) and caproate (C6:0) and contained negligible amounts of the transition fatty acids laurate (C12:0) and myristate (C14:0), which are typically high in coconut and palm oils and do not exhibit the same biochemical properties as the canonical MCFAs caproate (C6:0), caprylate (C8:0), and caprate (C10:0). Analysis of this ingredient through third-party testing gave the following analytical values: 51.4% caprylate (C8:0), 39.1% caprate (C10:0), <0.1% laurate (C12:0), and <0.01% each of all other fatty acids (Eurofins Nutrition Analysis Center, Des Moines, IA, USA). Caproate (C6:0) was not reported, but based on difference analysis, it is projected at approximately 8%. Since fish vary in their relative proportions of EPA (C20:5n3) and DHA (C22:6n3) [27–29], MEG-3^™^ 0355TG Oil (DSM Inc, Parsippany, NJ, USA) was chosen as the FO source of LCPUT(n3) as it is enriched for DHA (C22:6n3) over EPA (C20:5n3), with manufacturer-analyzed values of 36.5% DHA and 5% EPA.

MCT and FO were added to the foods at 7% and 2.85%, respectively, on a dry matter basis. Predicted composition based on certified analysis of ingredients and formulation levels indicated that the MCT-containing foods had 0.6% caproate (C6:0), 3.6% caprylate (C8:0), and 2.7% caprate (C10:0), while the FO-containing foods had 0.14% EPA (C20:5n3) and 1.0% DHA (C22:6n3), all on a dry matter basis (S2 Table). The MCT level was selected by choosing a dietary inclusion that would provide 30% of total dietary fat as MCT, which has been employed with companion animals [35, 36]. The FO level was targeted to be at a dietary inclusion level considered safe and high, as demonstrated by a published review of dietary FO levels employed in cats [37]. These levels have been observed to exert physiological effects and exert an impact on circulating levels of lipids.

### Study design and measurements

Animal care research technicians were blinded to the identity of the foods provided and also blinded to the group identity of cats for purposes of sample collection. In addition, all sample analysts were blinded to the group identity origin of samples during sample analysis. All cats were domestic short hair breed, were owned by the commercial funders of this research, and were either from on-site husbandry or acquired from licensed breeders. Sample size was based on effect sizes from a previous study [33] and was designed to achieve 80% power to detect a 20% difference between groups for selected lipids while factoring in the need for correction for multiple between-group testing and a typical subject dropout rate of 5%. During the washout period, all cats (N = 64) were fed the control food for 14 days. Cats were then randomized onto one of four diets (n = 16 each; CON, MCT, FO, FO+MCT) for 28 days by equitably distributing the cats into groups based on sex, weight, age, and group versus individual housing such that there were no significant differences in these parameters across groups by one-way ANOVA (*P* > 0.8 for all). Cats continued their preferred housing arrangement, as previously determined by colony veterinarian. Group- and individually housed cats were equally distributed across dietary groups, with 10 cats from each group being group-housed and 6 cats from each group individually housed with access to group social areas (S3 Table). All cats had continual access to electronic feeders which recorded daily food intake (g/day) for each cat. Fresh food was provided daily and feeders allowed access to food until individuals had consumed an amount calculated to maintain body weight; water was available ad libitum. On blood collection days, access to feeders was blocked overnight until completion of phlebotomy the following morning.

Plasma was collected for clinical blood chemistry and global metabolomics screening prior to consumption of test foods at the end of the washout period to serve as a baseline (one day before consuming test foods) and again at day 28 of feeding the test foods. Cats were fasted for 12 hours prior to plasma collection. The total amount of blood drawn was 11 ml. Clinical blood chemistry was performed on a COBAS c501 module (Roche Diagnostics Corporation, Indianapolis, IN, USA). Analysis of plasma metabolomics was performed by Metabolon (Morrisville, NC) as previously described [38, 39].

### Statistical analysis

Obtained metabolite values were natural log transformed, and statistics were performed on transformed values. Natural log-transformed metabolite values at baseline were subtracted from transformed values at day 28. This allowed for each cat to serve as its own control and for the reporting of the change in a given metabolite that was induced by the diet rather than a cross-sectional snapshot.

Difference values are presented as log_2_ fold change (log_2_FC) from baseline to day 28.

Changes from baseline across diets for the global plasma metabolome were assessed with the Metaboanalyst platform v4.0 [40]. Sparse partial least squares analysis (SPLS) was used to distinguish between diet groups (number of components = 2, validation method = 5-fold cross validation, number of predictors = 20), and Random Forest was used to detect metabolite predictors of group identity (number of trees = 2000, number of predictors = 20, Randomness = On).

The following statistical analyses were performed in JMP (Version 13.1–14.2. SAS Institute Inc., Cary, NC, 1989–2019): multivariate analysis of variance (MANOVA), one-way analysis of variance (ANOVA), post hoc analysis using Tukey’s honestly significant difference (HSD) test, and paired t-test. Determination of whether the change from baseline of a class of lipids or postbiotics differed across the diet groups was performed by MANOVA using the Identity function, which fits a model for each metabolite individually and then jointly tests the models together. The separate MANOVA *P* values for Wilks’ Lambda, Pillai’s Trace, Hotelling-Lawley, and Roy’s Max Root are reported in S4 Table. Only where *P* values for all of these metrics were less than 0.05 was the metabolite class considered significantly impacted by diet and allowed to proceed for further analysis. One-way ANOVA with Cauchy distribution was used to examine changes to individual metabolites within a class resulting from dietary oil intervention, with *P* values false discovery rate (FDR) adjusted according to the Benjamini and Hochberg procedure [41]. With ANOVA p values as an input, q values were generated for all metabolites in the R computing environment [42] using the “qvalue” function in the R package qvalue v2.14.1 [43]. Statistical significance was assigned to a metabolite when both of the following criteria were met: Benjamini and Hochberg FDR *P* ≤ 0.05 and q ≤ 0.1.

The sequential statistical process for testing whether a change from baseline for a given metabolite was significant, whether changes in individual metabolites constituting as class were different from zero within a group, and whether the changes from baseline for individual metabolites differed between groups was as follows: when MANOVA determined significance for a multivariate biochemical class, the delta for each metabolite in that class was subsequently assessed for a group effect by univariate ANOVA to determine which metabolites in that class that drove the significance observed for the class as a whole. Then, the degree to which a given metabolite exhibited a change from baseline within a group was assessed by paired t-test. Finally, the relative change from baseline for each metabolite was compared for all pairs of diet groups by Tukey’s HSD and reported using Connecting Letters.

## Results

### Characteristics of cats in the study

Sixty-four cats were randomized to one of four foods: CON, MCT, FO, or FO+MCT. The mean age was 5.7 years and mean weight was 5.2 kg; 37.5% were male (S3 Table). All cats except one were healthy as gauged by the colony veterinarian based on physical examination and clinical blood work. This cat was in the FO group and was diagnosed with hip dysplasia and managed hypertension. Participation in this trial was deemed in the best interest of the cat as FO-containing foods are used in veterinary practice to manage these conditions, and the FO test food contained therapeutic levels of FO similar to Hill’s Prescription Diet^®^ j/d, a food prescribed for mobility issues. One cat in the FO+MCT group did not complete the trial due to issues unrelated to the trial. Three additional cats (two in the MCT group, one in the FO+MCT group) completed the trial but were missing a plasma collection, which necessarily excluded them from a paired analysis for changes between baseline and week 4 of feeding. All cats returned healthy to the colony after the study.

### Impact of dietary oils on clinical blood chemistry

Clinical blood chemistry values did not exceed or diminish beyond normal ranges for healthy felines (S4 Table). FO consumption significantly decreased total triglycerides from baseline (delta = -10.8 mg/dL; *P* = 0.001), consistent with its known effects on blood lipids in other species [44]. Circulating cholesterol was decreased from baseline in the MCT, FO and FO+MCT dietary oil groups but unchanged in the control group. Average reductions from baseline were: MCT (delta = -23.7 mg/dL; *P* = 0.009), FO (delta = -10.3 mg/dL; *P* = 0.052), and FO+MCT groups (delta = -42.0 mg/dL; *P* < 0.001). These changes in cholesterol from baseline were significantly different among these three groups according to ANOVA, and the decrease induced by FO+MCT was significantly lower than the decrease for FO alone by Tukey’s HSD post hoc test.

### Impact of dietary oils on the global plasma metabolome

Metabolomic analysis performed on plasma samples taken from cats at baseline and week 4 identified 656 metabolites. Of these, one-way ANOVA showed that 456 metabolites were significantly different (Benjamini and Hochberg FDR *P* ≤ 0.05, q ≤ 0.1) across the 4 dietary groups. Individual paired t-tests by diet indicated that 108 metabolites changed in the CON group from baseline values (18 up, 90 down), 199 metabolites changed in the MCT group (34 up, 165 down), 265 metabolites changed in the FO group (106 up, 159 down) and 373 metabolites changed in the FO/MCT group (115 up, 258 down) (S4 Table). During the course of the study, cats continued their preferred housing arrangement (group versus individually housed) as previously determined by colony veterinarian. In order to assess if housing type influenced the circulating metabolome under investigation, all detected metabolites were compared between group and individually housed cats by independent t-test. Of the 656 metabolites detected, after false discovery correction for the multiple testing, there were only 7 metabolites that were significantly different between the individual- and group-housed cats at p < 0.05 and q < 0.1. Further, only 3 of these metabolites belonged to a class of metabolites reported here (3 metabolites in the sphingolipid and ceramide class). Thus, there was no meaningful effect of housing type on the circulating metabolome.

**Fig 1:**
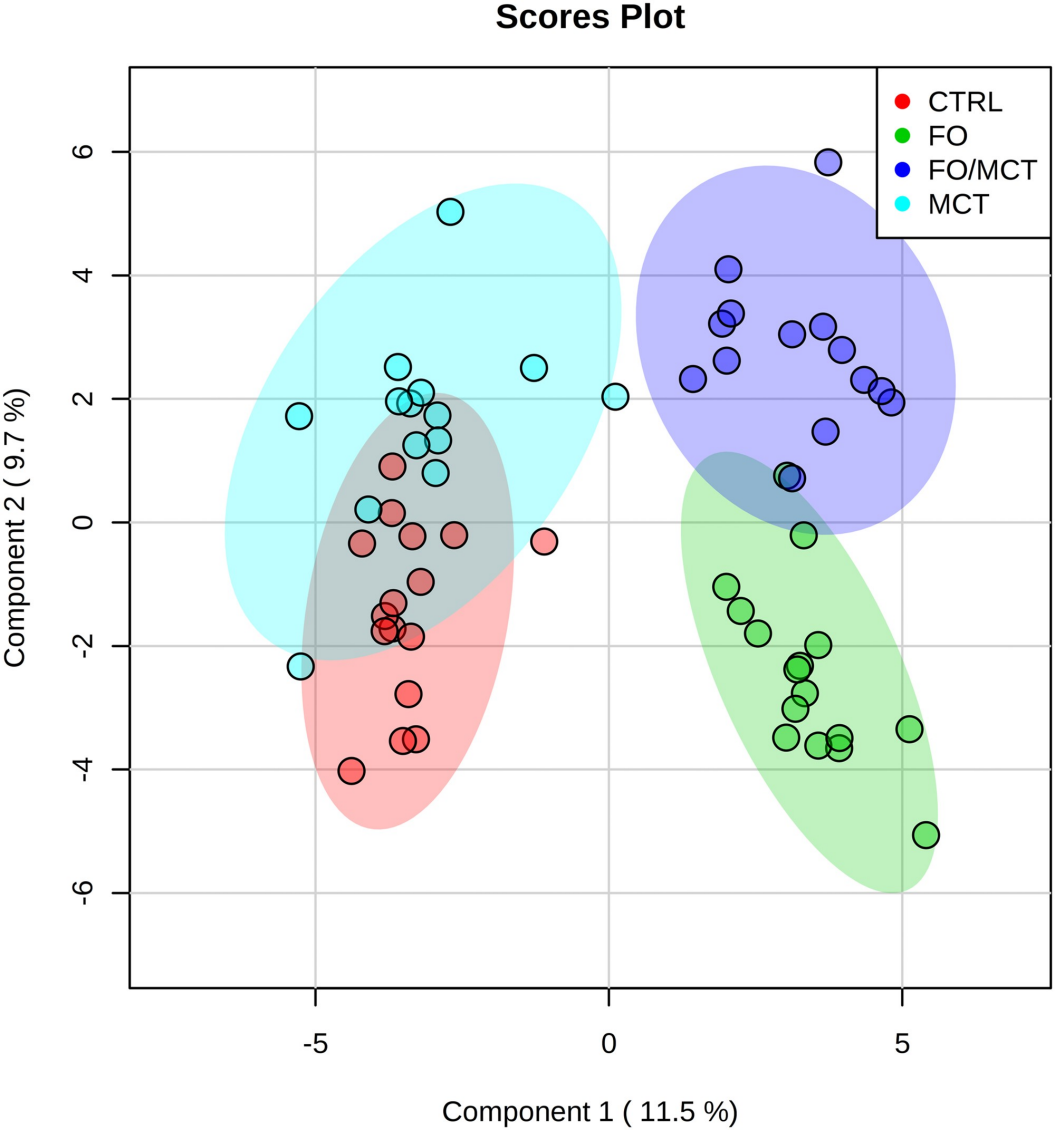
SPLS to distinguish among the foods tested. Component 1 segregated groups by the presence of FO in the food, and component 2 segregated groups by the presence of MCT in the food. Number of components = 2, validation method = 5-fold cross validation, number of predictors = 20. FO, fish oil; MCT, medium-chain fatty acid-containing triglycerides; SPLS, sparse partial least squares analysis.

Reduction of the high dimensionality of the dataset using SPLS indicated that there were differences among the groups’ changes from baseline (Fig 1), with greater separation from control by FO than by MCT feeding. SPLS loadings for components 1 and 2 are provided as supplemental information (S4 Table). Random Forest analysis provided discrimination between the dietary groups with an overall out of bounds class error of 8.3%, where 15/16 cats were correctly assigned to the control group (class error 6.3%), 12/14 cats were correctly assigned to the MCT group (class error 14%), 15/16 cats were correctly assigned to the FO group (class error 6.3%) and 13/14 cats were correctly assigned to the FO/MCT group (class error 7.1%) (S1 Fig). The only misclassification among CON and oil groups was between CON and MCT, as no cats consuming FO or FO/MCT were misclassified as CON or MCT. As expected, the top 20 ranked Random Forest predictors were lipid species (S1 Fig). Major lipid classes appearing in the metabolomics data set were chosen for further analysis: non-esterified fatty acids (NEFA), glycerophosphatidylcholines (GPCs), glycerophosphatidylinositols (GPIs), glycerophosphatidylethanolamines (GPEs), sphingolipids/ceramides, acylcarnitines, and N-acyl amino acids/neurotransmitters (NAAN). Additionally, given the recently noted impact of dietary lipids on the gut microbiome and justified by the statistical significance of multiple microbial metabolites by ANOVA (Benjamini and Hochberg FDR *P* ≤ 0.05, q ≤ 0.1), indoles and phenols were assessed for differences by food.

### Impact of MCT and FO on NEFAs

**Fig 2:**
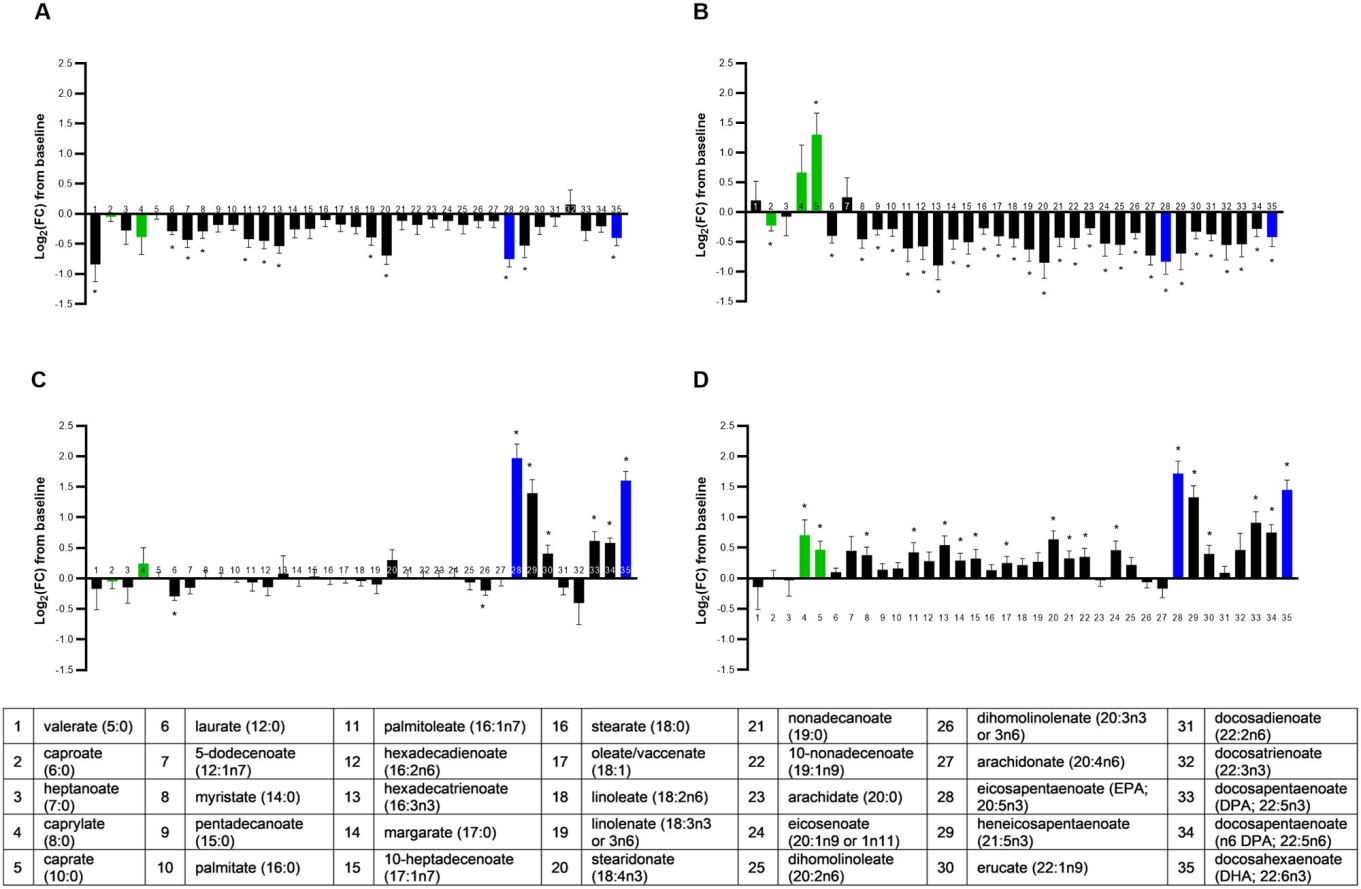
Change from baseline to day 28 (log_2_ fold change) in plasma levels of non-esterified fatty acids. Cats were fed A) control food, B) food with MCT, C) food with FO, or D) food with MCT and FO. Lipid metabolites are presented in order of increasing chain length and, within a chain length, by increasing unsaturation. Green bars indicate fatty acids found in the MCT ingredient; blue bars indicate fatty acids in the FO ingredient. FC, fold change; FO, fish oil; MCT, medium-chain fatty acid-containing triglycerides. *P < 0.05 compared with baseline.

The metabolite class of NEFAs was significantly different across groups in a multivariate manner (MANOVA *P* < 0.001; S4 Table) with a broad impact of dietary oil inclusion on plasma NEFAs; 86% (30/35) of the observed NEFA changes from baseline were significantly different across foods (ANOVA median *P* < 0.001). Out of the four food types, the FO+MCT group showed the most consistent increases in circulating NEFAs of all chain lengths and degrees of unsaturation (Fig 2). In contrast, the CON group remained largely unchanged, the MCT group exhibited broad decreases in almost all NEFAs, and the FO group showed very specific increases of the LCPUFA(n3) expected to occur with FO. Provision of FO+MCT shifted the response of NEFA levels from mainly decreases seen with MCT alone to mostly increases.

The MCFA caprate (C10:0) (bar 5 in Fig 2) was significantly different across groups by ANOVA (*P* ≤ 0.001), and while both the MCT and FO+MCT groups exhibited significantly increased levels of caprate (C10:0) from baseline, only the increase in the MCT group was significantly different than all other groups. Another MCFA, caproate (C6:0), differed by food (bar 2 in Fig 2) and significantly increased from baseline in the MCT group, but by pairwise assessment this change was not different from that observed for the other groups once Tukey’s HSD corrected for multiple group-wise testing. The remaining MCFA, caprylate (C8:0) (bar 4 in Fig 2), was not different across the foods although it was increased in the FO+MCT group alone. The changes from baseline for the transition fats laurate (C12:0) and myristate (C14:0) (bars 6 and 8 in Fig 2, respectively) were significantly different across the food groups. However, the predominant response was a decrease in these fatty acids, even in the MCT food group, which is consistent with their absence in the MCT oil employed in this study.

Since MCFAs are extensively converted to ketone bodies in the liver, circulating levels of the ketone body beta-hydroxybutyrate (BHBA) were examined in the metabolomics data. BHBA differed according to food group (*P* < 0.001) but only the FO+MCT group had a significant change from baseline, an increase in log_2_FC of 1.50 ± 0.18 (*P* < 0.001) which was numerically higher than the next highest group. The log_2_FC of BHBA in other groups did not reach significance: CON, -0.17 ± 0.18; MCT, 0.29 ± 0.19; FO, 0.38 ± 0.19. These data indicate that the addition of FO to MCT promoted the conversion of dietary MCFA to ketone bodies.

The following LCPUFA(n3) were strongly and significantly altered by food type: stearidonate (18:4n3), EPA (20:5n3), heneicosapentaenoate (21:5n3), docosapentaenoate (DPA, 22:5n3), and DHA (22:6n3) (bars 20, 28, 29, 33, and 35 in Fig 2, respectively). All these were significantly increased from baseline in both the FO and FO+MCT groups except stearidonate (18:4n3) in the FO group, and levels of these LCPUFAs were significantly higher than in the CON and MCT groups. The inclusion of MCT with FO did not impact accumulation of any of these LCPUFA, as they were not significantly different between the FO and FO+MCT groups.

With regards to n6 fatty acids, the n6 LCPUFA docosapentaenoate (n6 DPA, 22:5n6; bar 34 in Fig 2) was also increased from baseline in the groups that consumed FO or FO+MCT, and these groups exhibited levels significantly higher than the CON or MCT groups. Changes from baseline in arachidonic acid (C20:4n6; bar 27 in Fig 2), a precursor to proinflammatory lipid signaling mediators including prostaglandins, thromboxanes, and leukotrienes, was not affected by the LCPUFA(n3)-containing foods (FO, FO+MCT). However, the change from baseline of arachidonic acid (C20:4n6) did vary across the groups, which was entirely driven by a decrease in the MCT diet group.

### Impact of dietary oils on complex lipids

#### GPCs

**Fig 3:**
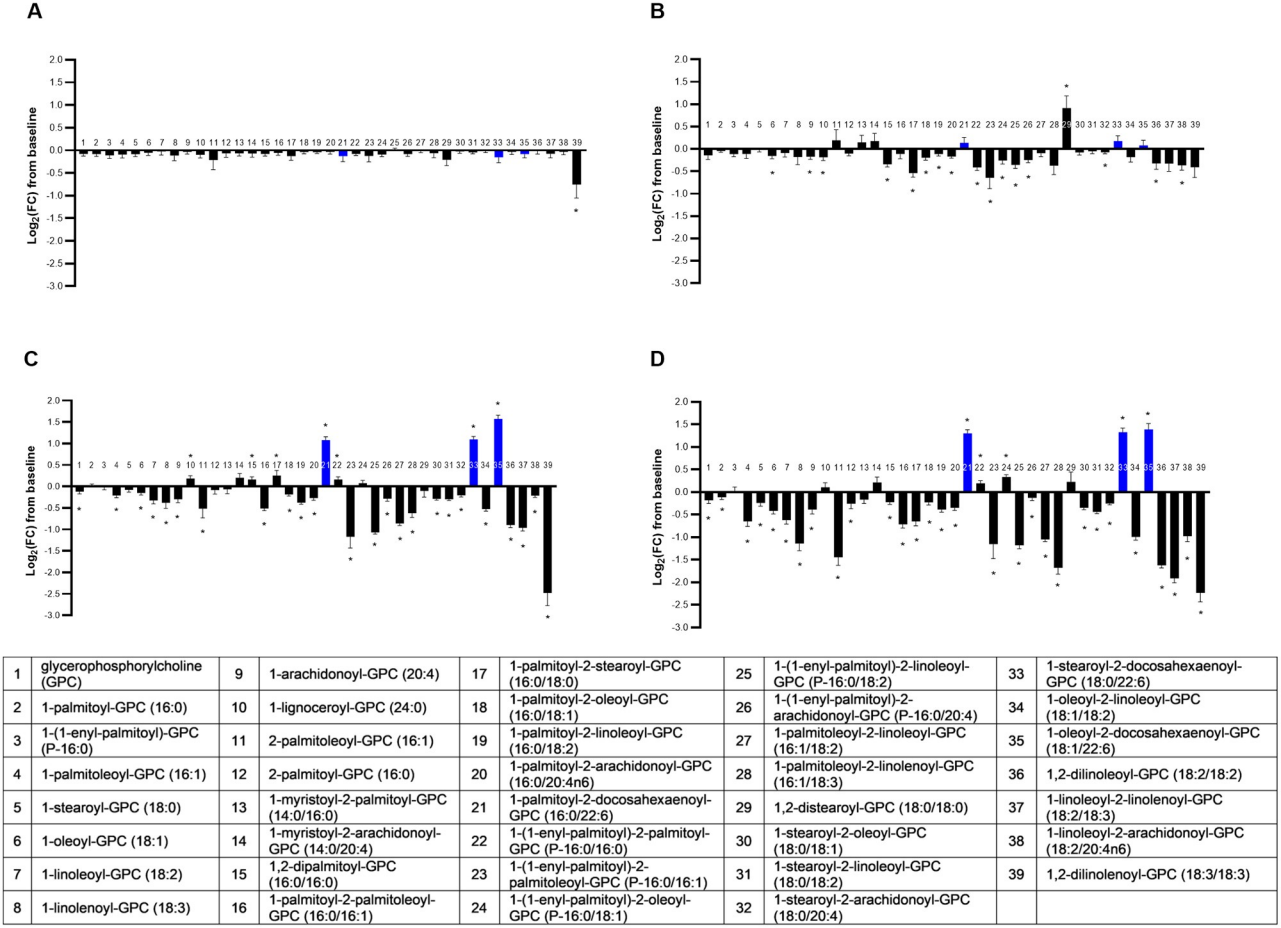
Change from baseline to day 28 (log_2_ fold change) in plasma levels of glycerophosphatidylcholines. Cats were fed A) control food, B) food with MCT, C) food with FO, or D) food with MCT and FO. Lipid metabolites are presented in order of increasing chain length and, within a chain length, by increasing unsaturation. Blue bars indicate those with a 22:6n3 chain. FC, fold change; FO, fish oil; MCT, medium-chain fatty acid-containing triglycerides. *P < 0.05 compared with baseline.

The metabolite class of GPCs was significantly different across food groups in a multivariate manner (MANOVA *P* < 0.001; S4 Table). Generally, there was a broad impact of dietary oil inclusion on GPC in that 87% (34/39) of observed GPC changes from baseline were significantly different across the groups (ANOVA median *P* < 0.001; Fig 3). This effect may have been slightly driven by reduced availability of GPC precursors. Choline differed significantly by food group, and although the CON and FO groups had no change from baseline, the MCT (log_2_FC -0.28 ± 0.07; *P* = 0.002) and FO+MCT groups (log_2_FC -0.29 ± 0.07; *P* < 0.001) had reduced choline levels relative to baseline. This response of choline in the MCT and FO+MCT groups was significantly different from that in the CON and FO groups. It is unlikely that this reduction in choline was due to a paucity of methylation donors, as an examination of trimethylglycine in the metabolomics dataset showed significant increases from baseline in the MCT (log_2_FC 0.40 ± 0.09; *P* < 0.001) group and to a lesser degree in the FO (log_2_FC 0.20 ± 0.09; *P* = 0.05) and FO+MCT (log_2_FC 0.37 ± 0.12; *P* = 0.01) groups. There were weakly significant decreases from baseline of glycerophosphocholine for both the FO (log_2_FC -0.12 ± 0.05; *P* = 0.02) and FO+MCT (log_2_FC -0.18 ± 0.07; *P* = 0.02) groups.

In contrast to the broad decreases of NEFAs in the MCT group, the MCT effects on GPCs were less pronounced. Another response-type differentiating NEFAs from GPCs was exhibited by FO, which produced highly specific effects on a select few NEFAs but had a broad impact on GPCs. The combination of FO+MCT produced an effect on GPCs that was as pervasive as for NEFAs, but the predominant changes for GPCs were decreases in this metabolite class but increases in NEFAs. The MCT food produced one of the few observed increases in a GPC containing two saturated fatty acids; 1,2-distearoyl-GPC (18:0/18:0; bar 29 in Fig 3) differed across foods, and this effect was driven by the MCT food group. This increase was not observed in the FO+MCT group, perhaps due to a moderating impact of FO incorporation of saturated fatty acids into phospholipids. In contrast, both of the other di-saturated fatty acid-containing GPCs observed in the data set (1,2-dipalmitoyl-GPC [16:0/16:0; bar 15 in Fig 3] and 1-palmitoyl-2-stearoyl-GPC [16:0/18:0; bar 17 in Fig 3]) were significantly decreased by MCT food. None of the 18:0-containing GPC with an unsaturated fatty acid present at position 2 were increased by the MCT food. The presence of a saturated fatty acid at position 2 of GPC is less common than an unsaturated fatty acid at this position. This result may indicate a specific effect of MCT to increase 1,2-distearoyl-GPC (18:0/18:0).

Although the CON food had no impact on any arachidonate (C20:4n6)-containing GPC, 5 out of 6 GPCs detected in the metabolomics dataset that contain this proinflammatory n6 LCPUFA were affected by food type, with the MCT, FO, and FO+MCT diets all decreasing these C20:4n6-containing GPCs from baseline. The only C20:4n6-containing GPC observed in the metabolomics dataset that was not decreased by the MCT, FO, or FO+MCT foods was 1-myristoyl-2-arachidonoyl-GPC (1-C14:0-2-C20:4n6-GPC; bar 14 in Fig 3). GPCs containing FO-derived EPA (20:5n3) were not detected in the metabolomics dataset. However, levels of three DHA (22:6n3)-containing GPCs were significantly increased from baseline by both FO and FO+MCT foods (bars 21, 33, and 35 in Fig 3) but were not changed by the CON or MCT foods.

#### GPIs

**Fig 4:**
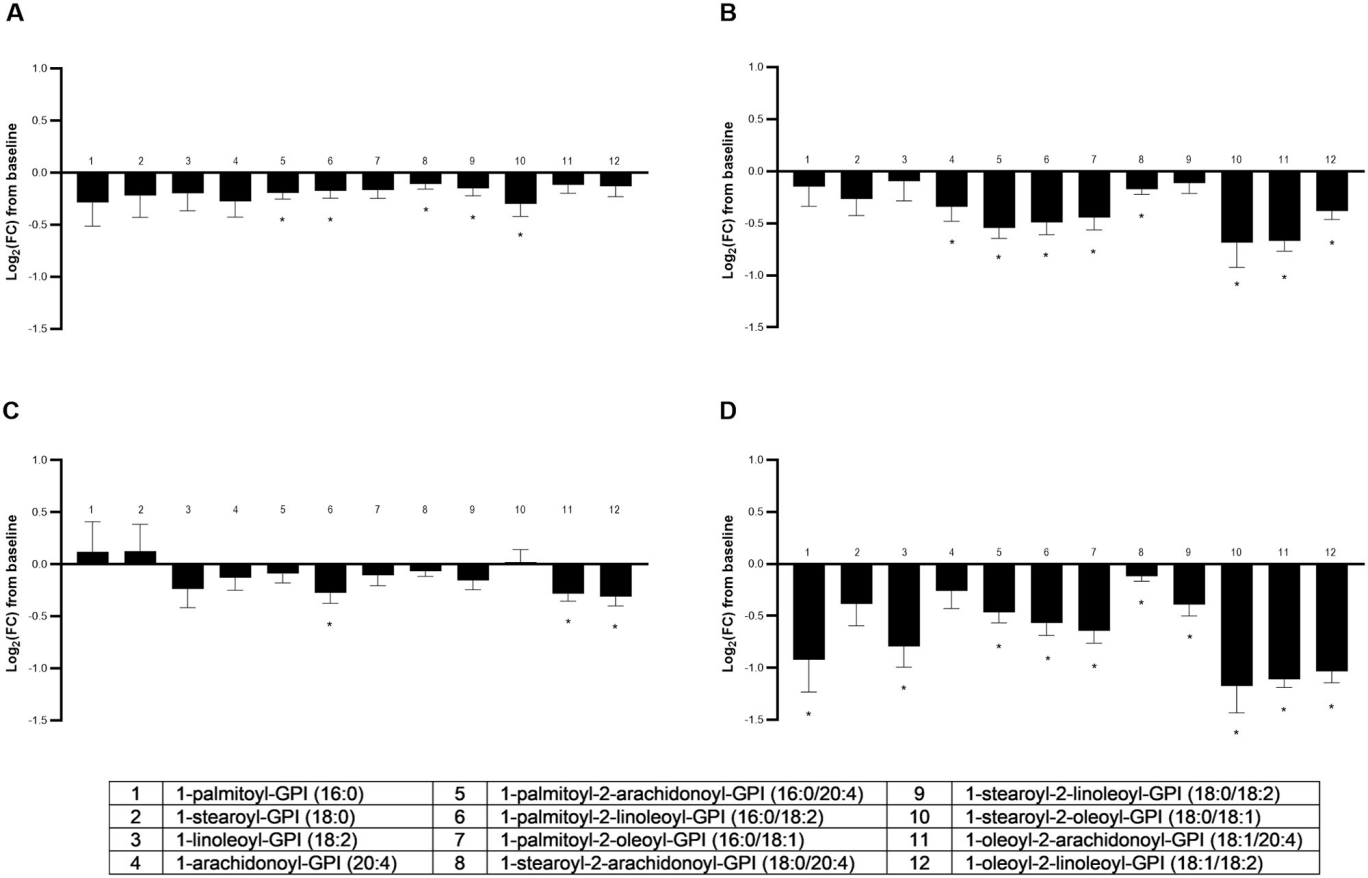
Change from baseline to day 28 (log_2_ fold change) in plasma levels of glycerophosphatidylinositols. Cats were fed A) control food, B) food with MCT, C) food with FO, or D) food with MCT and FO. Lipid metabolites are presented in order of increasing chain length and, within a chain length, by increasing unsaturation. FC, fold change; FO, fish oil; MCT, medium-chain fatty acid-containing triglycerides. *P < 0.05 compared with baseline.

The metabolite class of GPIs was significantly different across groups in multivariate fashion (MANOVA *P* < 0.001; S4 Table). Food type differentially affected 83% (10/12) of observed changes from baseline in GPCs (ANOVA median *P* < 0.001) (Fig 4). Only decreases from baseline were observed for all groups; no GPI significantly increased. This effect could have been driven in part by changes in the precursor inositol. However, myoinositol was the only circulating inositol detected, and although changes from baseline were weakly significantly impacted by food type, only the CON diet showed a significantly change (log_2_FC 0.35 ± 0.11; *P* = 0.01). This change was not significantly different than the MCT, FO, and FO+MCT food groups.

The most pronounced effect on GPIs was seen in the FO+MCT group, with 10/12 GPIs significantly changed from baseline. This may have been largely due to the presence of MCT, as that food group produced a change from baseline for 8/12 GPIs, whereas the FO diet gave changes in only 3/12 GPIs. Further indicating the influence of MCT intervention, the MCT-only food reduced all 4 detected arachidonate (C20:4n6)-containing GPIs from baseline and the FO+MCT group reduced 3 of these, while the FO intervention only reduced one. No EPA (C20:5n3) or DHA (C22:6n3)-containing GPIs were detected in the metabolomics data set.

#### GPEs

**Fig 5:**
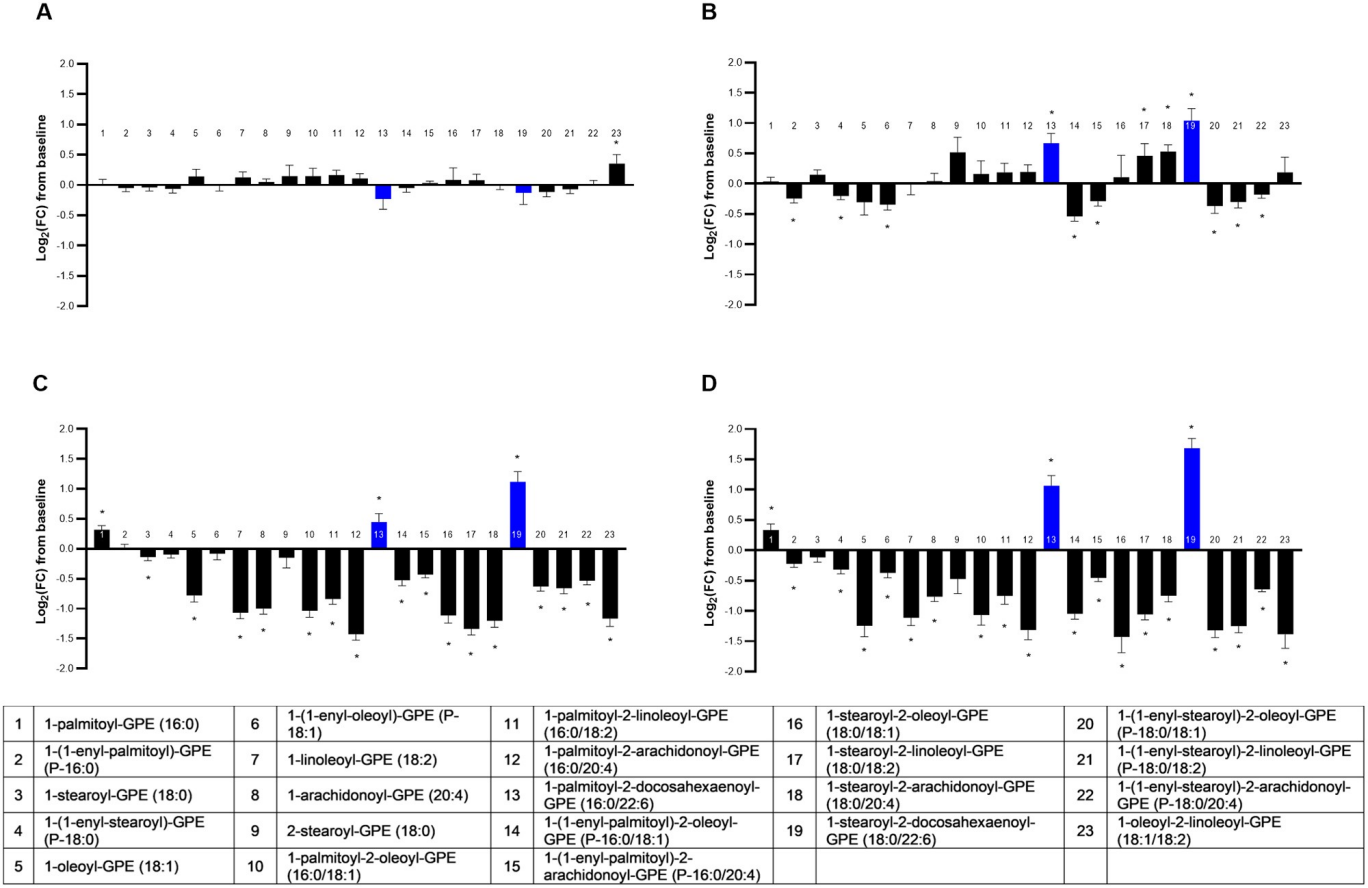
Change from baseline to day 28 (log_2_ fold change) in plasma levels of glycerophosphatidylethanolamines. Cats were fed A) control food, B) food with MCT, C) food with FO, or D) food with MCT and FO. Lipid metabolites are presented in order of increasing chain length and, within a chain length, by increasing unsaturation. Blue bars indicate those that were strongly increased from baseline in all dietary oil intervention groups. FC, fold change; FO, fish oil; MCT, medium-chain fatty acid-containing triglycerides. *P < 0.05 compared with baseline.

GPE as a metabolite class was significantly different across groups in a multivariate manner (MANOVA *P* < 0.001; S4 Table). There was a broad impact of dietary oil inclusion on GPE relative to the CON group, with 96% (22/23) of the observed GPEs exhibiting changes that differed significantly across food types (ANOVA median *P* < 0.001; Fig 5). Nearly all significant changes in GPE were reductions from baseline levels, particularly in the FO and FO+MCT groups. This effect could have been perhaps driven in part by changes in precursor glycerophosphoethanolamine and phosphoethanolamine, the two ethanolamines detected in the metabolomics dataset, in the foods containing MCT and/or FO. Phosphoethanolamine decreased from baseline in the MCT (log_2_FC -0.27 ± 0.11; *P* = 0.03) and FO+MCT (log_2_FC -0.37 ± 0.15; *P* = 0.03) groups, and these changes were significantly different than the change from baseline observed for the CON group (log_2_FC 0.44 ± 0.10; *P* < 0.001). Furthermore, glycerophosphoethanolamine decreased from baseline in the FO (log_2_FC -0.15 ± 0.04; *P* < 0.001) and FO+MCT groups (log_2_FC -0.20 ± 0.0.06; *P* = 0.01). Taken together, the most consistent decreases in ethanolamine precursors were in the FO+MCT group.

Consistent with heightened impact of FO+MCT on ethanolamine precursors, the most pronounced effect on GPEs was in the FO+MCT group; 21/23 GPEs significantly changed from baseline. The group consuming FO produced a change from baseline for 19/23 GPEs, whereas MCT induced a change in 12/23 GPEs. Of all the phospholipid classes of metabolites (GPC, GPI, GPE), the FO+MCT food exhibited its greatest magnitude of effects in the GPI and GPE series, with respective median log_2_FC of -0.72 ± 0.11 and -0.77 ± 0.18 compared with -0.40 ± 0.14 for GPC.

Although the CON food produced no changes from baseline for any arachidonate (C20:4n6)-containing GPEs, all five observed GPEs that contain this proinflammatory LCPUFA(n6) were affected by food group, driven largely by decreases in the FO and FO+MCT groups. The MCT food decreased two of the five C20:4n6-containing GPEs from baseline and increased one of the five (1-stearoyl-2-arachidonoyl-GPE [1-C18:0-2-C20:4n6-GPE]).

GPEs containing EPA (20:5n3) were not detected in this metabolomics dataset. However, two DHA (22:6n3)-containing GPE were observed in the metabolomics dataset: 1-palmitoyl-2-docosahexaenoyl-GPE (16:0/22:6n3) and 1-stearoyl-2-docosahexaenoyl-GPE (18:0/22:6n3) (bars 13 and 19 in Fig 5). Both of these 22:6n3-containing GPEs were strongly increased from baseline by all dietary oil intervention groups (MCT, FO, and FO+MCT). It was unexpected that the MCT food would increase 22:6n3-containing GPEs, and the magnitude of effect for MCT was as great as for the FO group. Thus, an additive effect of MCT and FO likely led to the FO+MCT group having the largest increases in the two 22:6n3-containing GPEs; the change from baseline for the FO+MCT group was about 50% greater than for the FO or MCT alone groups.

#### Sphingolipids and ceramides

**Fig 6:**
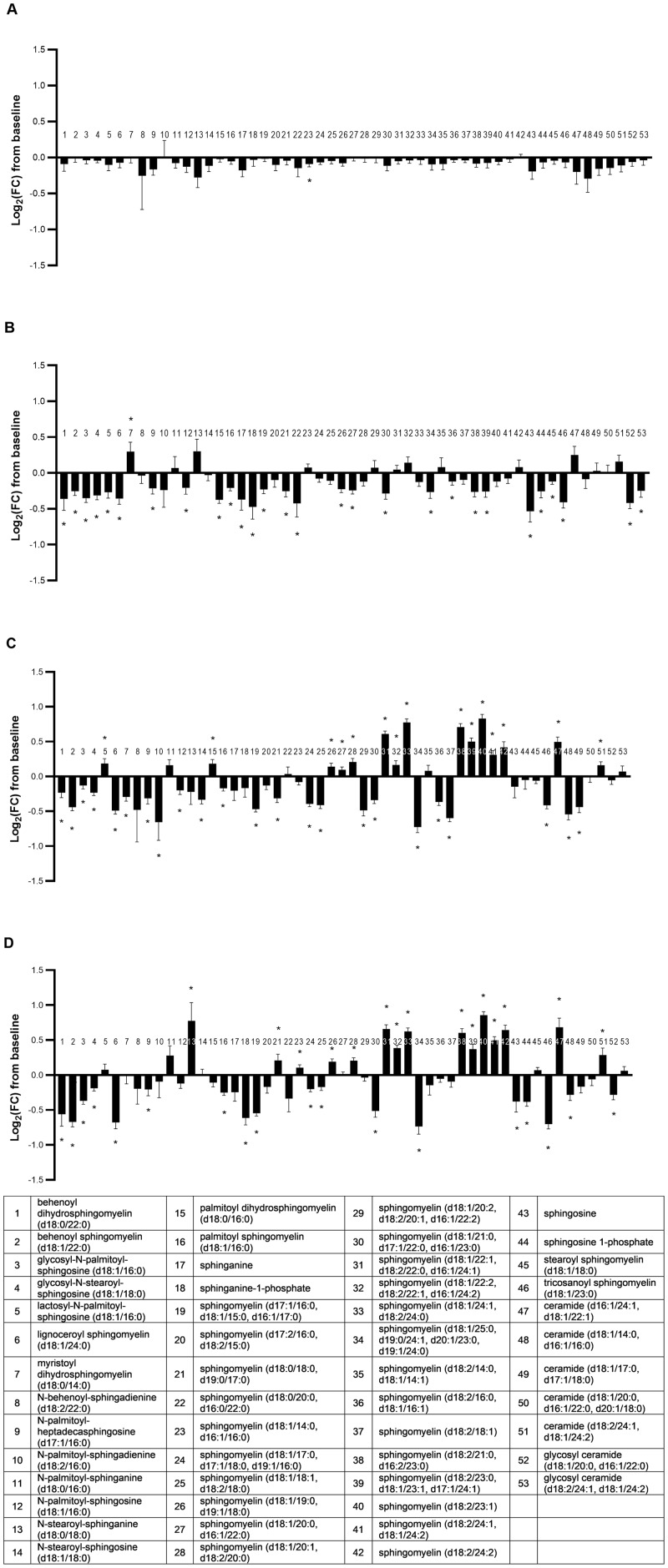
Change from baseline to day 28 (log_2_ fold change) in plasma levels of sphingolipids and ceramides. Cats were fed A) control food, B) food with MCT, C) food with FO, or D) food with MCT and FO. Lipid metabolites are presented in order of increasing chain length and, within a chain length, by increasing unsaturation. FC, fold change; FO, fish oil; MCT, medium-chain fatty acid-containing triglycerides. *P < 0.05 compared with baseline.

The metabolite class of sphingolipids and ceramides significantly differed across groups in multivariate fashion (MANOVA *P* ≤ 0.01; S4 Table). There was a highly significant and broad effect of dietary oil inclusion on individual sphingolipids and ceramides relative to the CON group, with 83% (44/53) of observed changes from baseline significantly affected by diet (ANOVA median *P* < 0.001; Fig 6). As opposed to the effects of the FO and FO+MCT diets on phospholipid metabolite classes (GPC, GPI, GPE), where the predominant change was a decrease from baseline levels, there was an mixture of both upward and downward shifts in sphingolipids and ceramides when cats consumed the FO or FO+MCT foods. The FO and FO+MCT groups had 12 of their increases and 14 of their decreases from baseline in common. Particularly, both FO and FO+MCT foods significantly changed the same 10/11 sphingolipids and ceramides containing a 24-carbon chain moiety with the same directionality to these changes; 8/10 of these changes were increases. However, the MCT group produced mostly downward shifts in sphingolipid and ceramide levels from baseline (only one metabolite in this class was significantly increased, while 28 were significantly decreased) in a manner consistent with its effects on the phospholipid metabolite classes. There were a total of 11/53 common metabolites in this class that were decreased in all three intervention groups (MCT, FO, FO+MCT), but none was increased in all three groups. It may be that the effects of MCT to decrease sphingolipids and ceramides were due to reductions in precursor metabolites, as MCT significantly decreased 4/4 detected precursors (sphinganine, sphingosine, and their respective phosphorylated congeners). The reduction in sphingolipid and ceramide precursors largely carried over into the FO+MCT food as all of these except sphinganine also decreased in the cats consuming this combination of oils; however, there was no change in these sphingolipid precursors in the CON or FO groups.

#### Acylcarnitines

**Fig 7:**
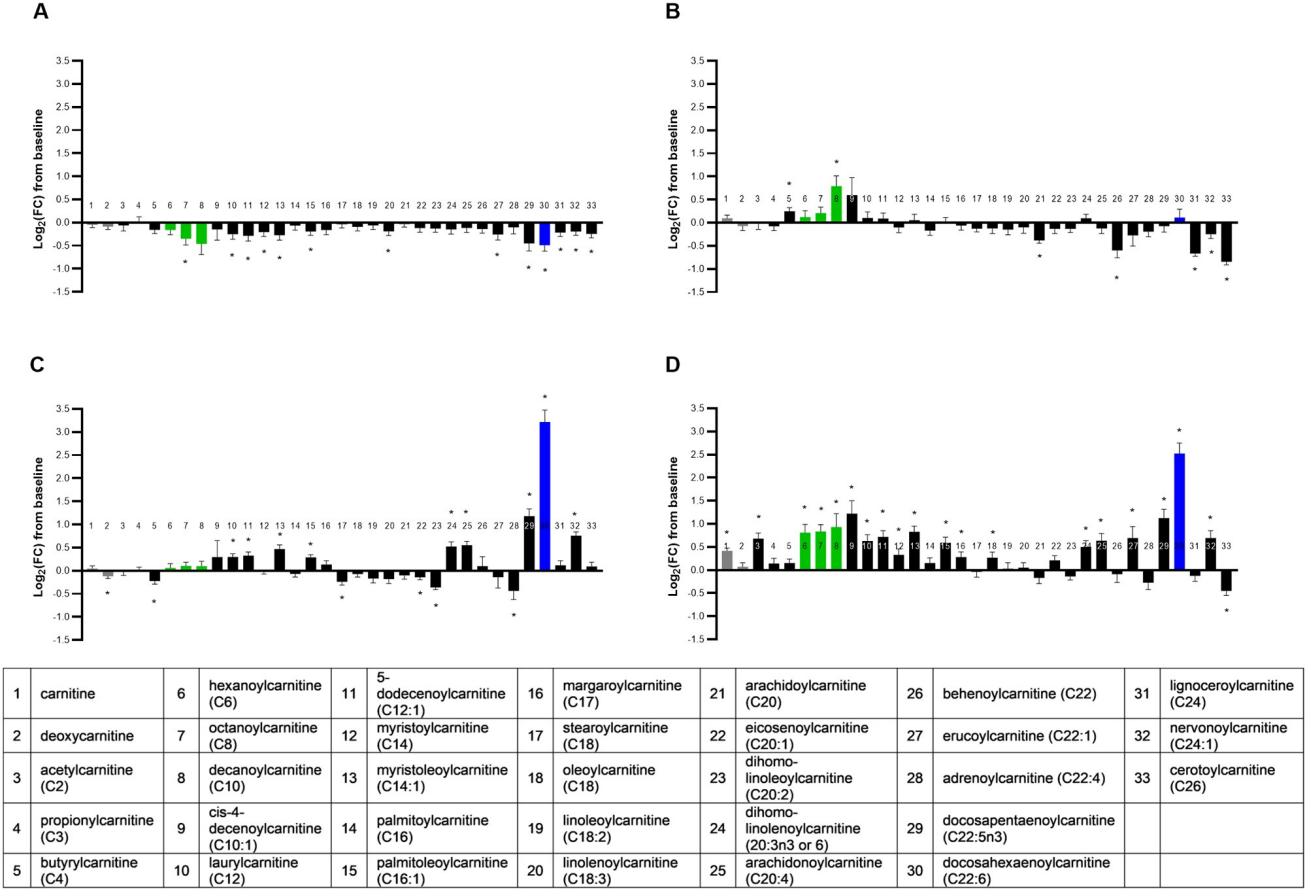
Change from baseline to day 28 (log_2_ fold change) in plasma levels of acylcarnitines. Cats were fed A) control food, B) food with MCT, C) food with FO, or D) food with MCT and FO. Lipid metabolites are presented in order of increasing chain length and, within a chain length, by increasing unsaturation. Gray bars indicate unacylated carnitine precursors to acylcarnitines, green bars indicate the MCFA-carnitines, and blue bars indicate LCPUFA(n3) carnitines. FC, fold change; FO, fish oil; MCT, medium-chain fatty acid-containing triglycerides. *P < 0.05 compared with baseline.

The metabolite class of acylcarnitines was significantly different across food groups in a multivariate manner (MANOVA *P* < 0.001; S4 Table), with 76% (25/33) of detected acylcarnitines altered by diet (ANOVA median *P* < 0.001; Fig 7). There were relatively few changes in acylcarnitines with the MCT food. Relative to transition and long-chain fatty acids, decanoate (C10:0), a dominant fatty acid in the MCT food, is proportionally more capable of being catabolized to energy without the strict requirement for conjugation to carnitine. Despite this, decanoylcarnitine (C10:0; bar 8 in Fig 7) strongly increased following consumption of the MCT food. A strong increase in decanoylcarnitine (C10:0) was also observed in the FO+MCT group, but not in the FO or CON groups, indicating that the driver of increased decanoylcarnitine (C10:0) is the presence of C10:0 MCFA in the MCT ingredient used to produce the MCT and FO+MCT foods.

Although the MCT food increased decanoylcarnitine (C10:0), the combination of FO+MCT increased the entire MCFA-carnitine series (hexanoylcarnitine [C6], octanoylcarnitine [C8], and decanoylcarnitine [C10; bars 6, 7, and 8 in Fig 7]) while MCT alone did not. Neither the FO alone nor CON foods produced this effect.

Consumption of MCT also led to highly significant reductions of relatively large magnitude for very long-chain saturated-acylcarnitines. The series of arachidoylcarnitine (C20), behenoylcarnitine (C22), lignoceroylcarnitine (C24), and cerotoylcarnitine (C26) (bars 21, 26, 31, and 33 in Fig 7) were all decreased by MCT consumption but were unaffected by FO and only the latter two were impacted in the CON group with a smaller magnitude of effect. Of note, the carryover of this effect to the FO+MCT group was not pervasive. Of this series of very long chain saturated-acylcarnitines, only the longest (cerotoylcarnitine [C26]) was decreased when FO was additionally present (FO+MCT food), indicating the modulating impact of FO on MCT provision.

Considering the effect of FO on acylcarnitines, the FO food increased several PUFA-carnitines, and this effect was also present in the FO+MCT food-fed cats. EPA (20:5n3)-carnitine was not observed in the metabolomics dataset, but dihomo-linolenoylcarnitine (20:3n3 or 6), docosapentaenoylcarnitine (C22:5n3), docosahexaenoylcarnitine (C22:6n3) and nervonoylcarnitine (C24:1) (bars 24, 29, 30, and 32 in Fig 7, respectively) were all significantly increased in both the FO and FO+MCT groups, in line with the availability of the precursor fatty acids of these carnitine conjugates being present in these foods. Both FO and FO+MCT consumption led to significant increases in arachidonoylcarnitine (C20:4) (bar 25 in Fig 7), although this carnitine conjugate of a proinflammatory fatty acid was unchanged in the MCT or CON groups. It is notable that the magnitude of effect to increase the aforementioned LCPUFA-carnitine conjugates was quite similar in both the FO and FO+MCT diet groups, which indicates that the presence of MCT did not appreciably impact the effect of FO on these lipid conjugates.

#### NAANs

**Fig 8:**
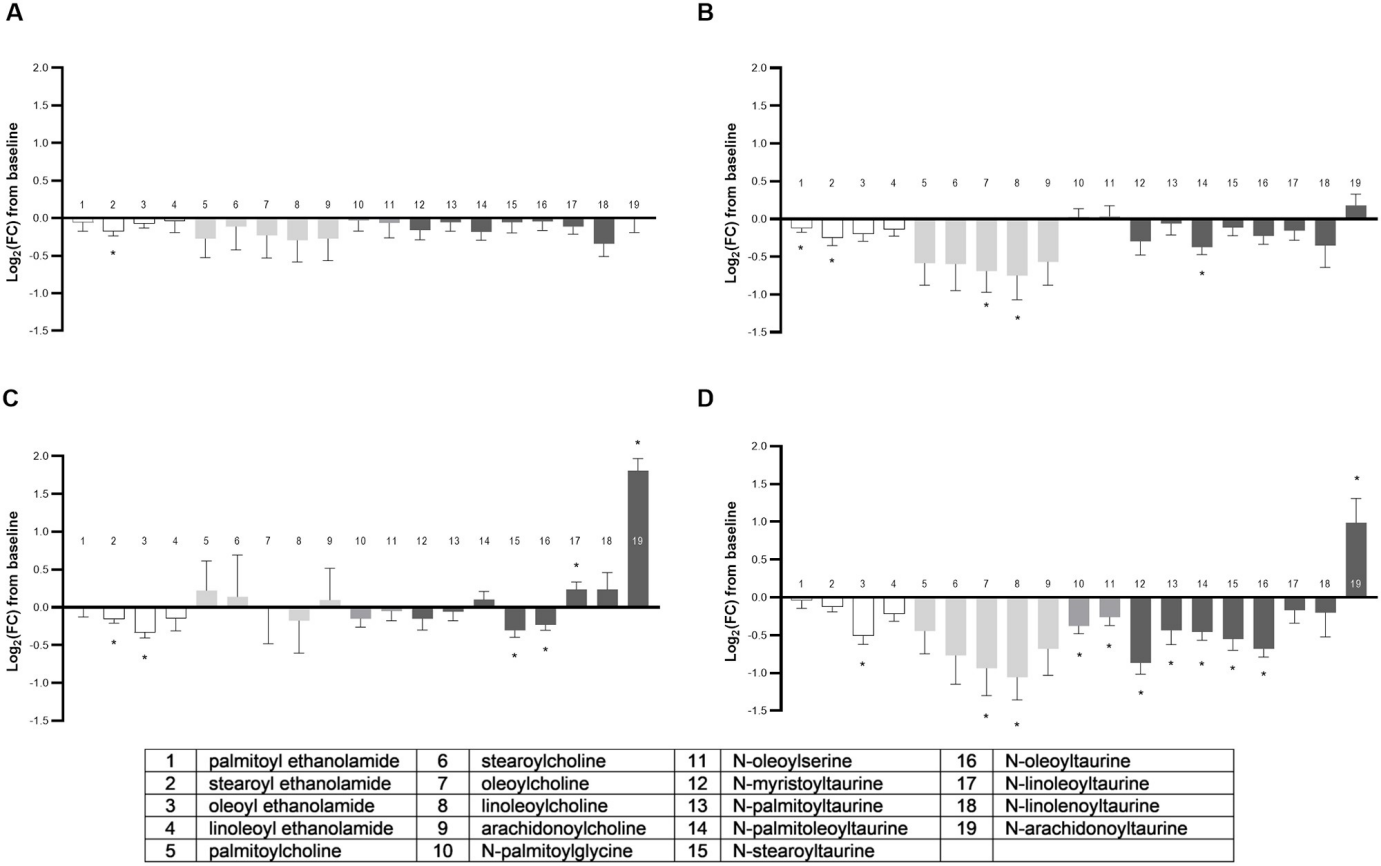
Change from baseline to day 28 (log_2_ fold change) in plasma levels of NAAN. Cats were fed A) control food, B) food with MCT, C) food with FO, or D) food with MCT and FO. White bars indicate the ethanolamide-type, light gray the choline-type, medium gray the amino acid-type, and dark gray the taurine-type of NAAN. FC, fold change; FO, fish oil; MCT, medium-chain fatty acid-containing triglycerides; NAAN, N-acyl amino acids and neurotransmitters. *P < 0.05 compared with baseline.

The metabolite class of NAANs was significantly different across groups in multivariate fashion (MANOVA *P* < 0.001; S4 Table) with 19 total metabolites detected and 58% (11/19) altered by food type (ANOVA median *P* = 0.0002; Fig 8). The combination FO+MCT food had significant changes from baseline in 58% (11/19) of NAANs. Further, 4/19 of those changed in the FO+MCT group were not produced in either FO or MCT oil alone, indicating a synergistic effect of the combination. The predominant changes from baseline in all four food types were decreases, with only three significant increases from baseline in any of the oil intervention foods.

Of the four ethanolamide-type of NAANs detected, two were decreased by MCT consumption (palmitoyl ethanolamide and stearoyl ethanolamide; bars 1 and 2 in Fig 8) and these contained a saturated acyl group. FO also produced a decrease in two ethanolamide-type NAANs (stearoyl ethanolamide and oleoyl ethanolamide), while FO+MCT decreased only oleoyl ethanolamide.

There were five choline-type NAANs detected, two of which were decreased by intervention with MCT (oleoylcholine and linoleoylcholine; bars 7 and 8 in Fig 8). This effect was also present in the FO+MCT combination group, but the FO intervention had no significant effect on choline-type NAANs, indicating that MCT provision was a driver of the effect on acylcholines.

In the amino acid-type NAANs, only the combination of FO+MCT showed changes from baseline, with decreases in both of the detected N-acyl-amino acids N-palmitoylglycine and N-oleoylserine, bars 10 and 11 in Fig 8, indicating an unanticipated synergistic effect.

The taurine-type NAANs manifested the greatest disparity in number of observed changes among dietary oil intervention groups. Whereas 1/8 taurine-type NAAN was decreased by the MCT food, 4/8 changed with the FO treatment (two decreased and two increased) and 6/8 were altered with the combination of FO+MCT (five decreased and one increased). Perhaps the most interesting change was an increase in the taurine-type NAAN conjugated to the arachidonoyl acyl group (N-arachidonoyltaurine) (bar 19 in Fig 8). Intervention with FO alone or FO+MCT, but not MCT alone, significantly increased N-arachidonoyltaurine. However, the magnitude of the log_2_FC increase was twice as large in the FO group (1.81 ± 0.16) as it was in the FO+MCT combination group (0.99 ± 0.32), suggesting that MCT had an ameliorating effect on N-arachidonoyltaurine induction by FO. It is perhaps surprising that FO and the FO+MCT diets led to an increase in N-arachidonoylcarnitine and N-arachidonoyltaurine but did not increase any other arachidonoyl (C20:4n6)-containing lipids among any of the metabolite classes examined here. Unlike N-arachidonoylcarnitine and N-arachidonoyltaurine, arachidonate-containing NEFA, GPC, GPI, and GPE are substrates for production of pro-inflammatory prostaglandins, leukotrienes, and thromboxanes and were unaltered by either FO or FO+MCT.

### Impact on microbial postbiotics

#### Indoles/indolic sulfates

**Fig 9:**
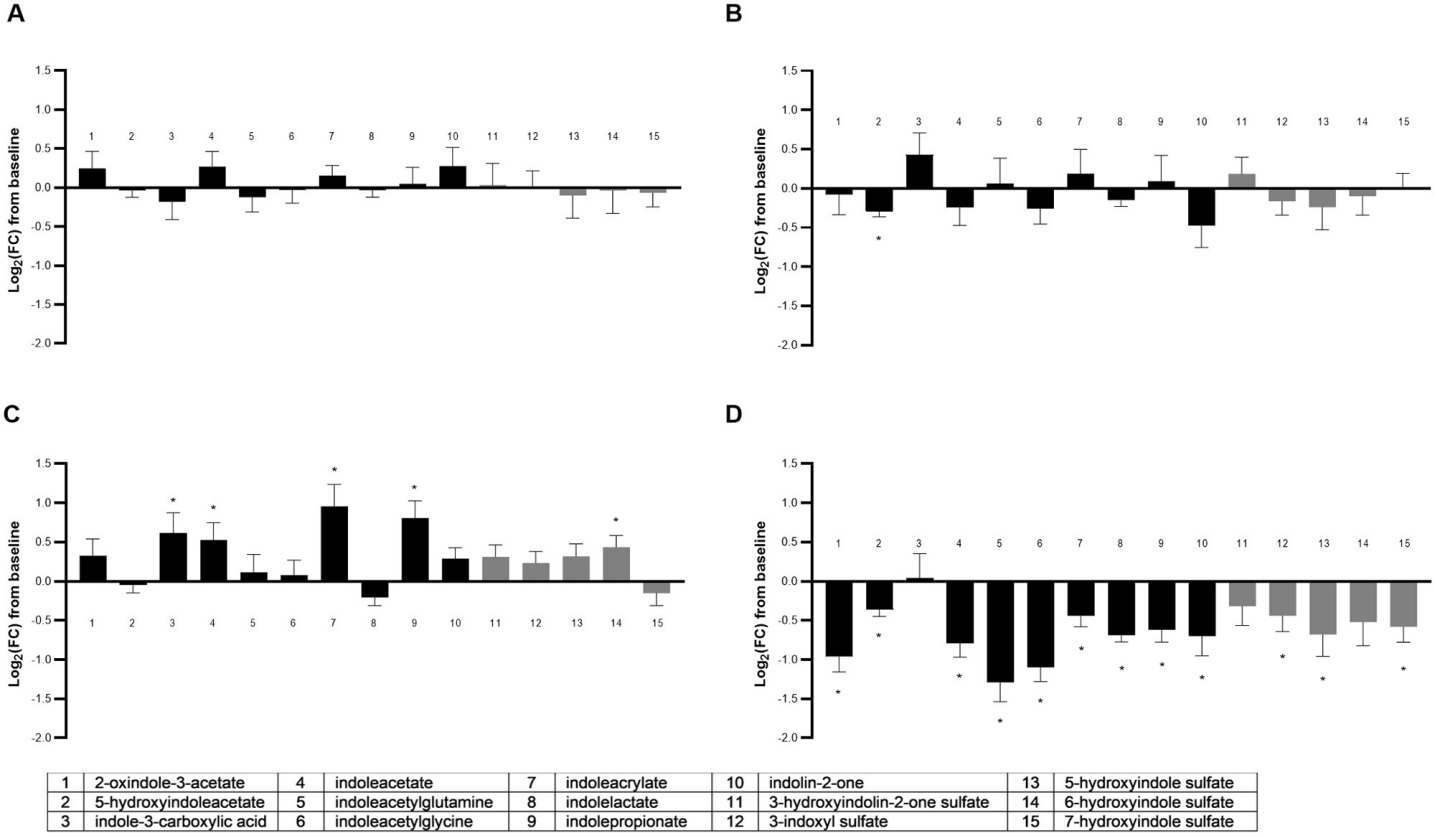
Change from baseline to day 28 (log_2_ fold change) in plasma levels of postbiotic indoles/indolic sulfates. Cats were fed A) control food, B) food with MCT, C) food with FO, or D) food with MCT and FO. Gray bars indicate the sulfated metabolites. FC, fold change; FO, fish oil; MCT, medium-chain fatty acid-containing triglycerides. *P < 0.05 compared with baseline.

The metabolite class of indoles and indolic sulfates was significantly different across groups in a multivariate manner with 15 total metabolites detected (MANOVA *P* < 0.001; S4 Table), 73% (11/15) of which were altered by food type (ANOVA median *P* < 0.001; Fig 9). Most (80%; 12/15) of the detected indoles and indolic sulfates were altered by the combination food FO+MCT, and all were decreases. In the FO+MCT group, there were decreases in not only indoles but also in the host-conjugated sulfated indoles. These decreases in the FO+MCT group did not appear to be derived from additive effects of the separate oils since the MCT group showed a change in only one of these metabolites, and the five changes in the FO group were all increases. Thus, there is an unanticipated and synergistic effect of FO+MCT combination food on indole postbiotics.

#### Phenols/phenolic sulfates

**Fig 10:**
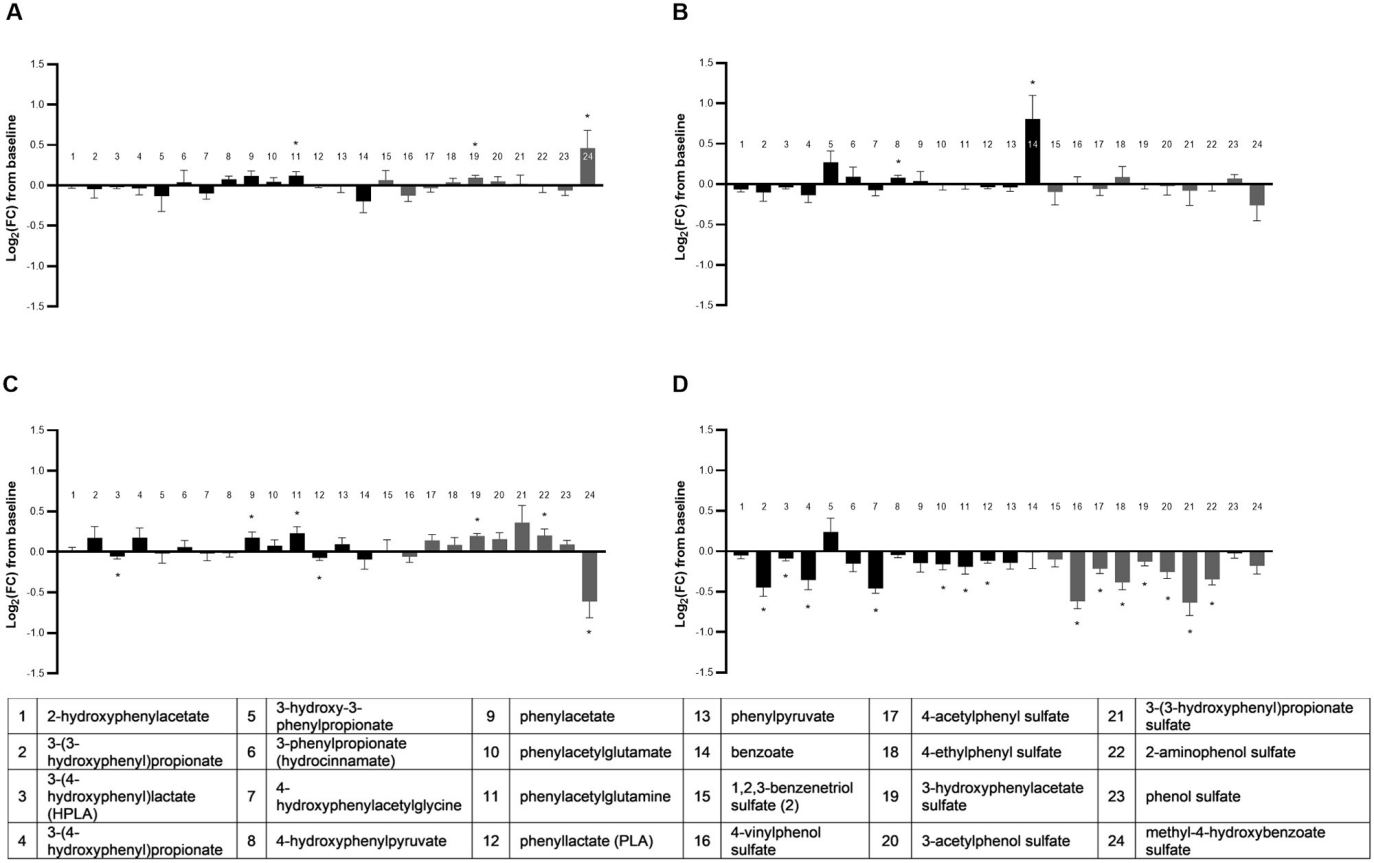
Change from baseline to day 28 (log_2_ fold change) in plasma levels of postbiotic phenols/phenolic sulfates. Cats were fed A) control food, B) food with MCT, C) food with FO, or D) food with MCT and FO. Gray bars indicate the sulfate-containing metabolites. FC, fold change; FO, fish oil; MCT, medium-chain fatty acid-containing triglycerides. *P < 0.05 compared with baseline.

The class of phenols and phenolic sulfates was significantly different across groups by multivariate analysis (MANOVA *P* < 0.001; S4 Table), with 24 total metabolites detected and 83% (20/24) altered by food (ANOVA median *P* = 0.0002; Fig 10). Similar to the indoles/indolic sulfates, the phenols and phenolic sulfates were disproportionately impacted by the combination FO+MCT food (14/24 changes from baseline) compared with the other food types and all were decreases. In contrast, the significant MCT-induced changes were both increases, and the changes in the FO group were split between increases and decreases. Therefore, there is a similar synergistic effect of FO+MCT combination food on phenol/phenolic sulfate postbiotics as for the indoles/indolic sulfate postbiotic metabolites.

## Discussion

This trial assessed the effects of two very different types of fats (long chain polyunsaturated versus medium chain saturated) and used global metabolomics to survey the levels of several classes of lipid metabolites: non-esterified fatty acids as well as the structural (GPC, GPI, GPE), metabolically active (acylcarnitines), and biologically active (NAAN) complex lipids into which these fats are incorporated. The effect of these dietary fats on circulating microbial postbiotics was also examined given recent work demonstrating the effects of dietary lipids on microbiome composition. While the level of MCT was twice the level of FO in the current study, that is typical and due to their desired characteristics; in general, dietary FO is used at levels much lower than MCT. Comparison of identical dietary levels of FO and MCT is not an accurate representation of the use of these fats in nutrition; fatty acids from FO exert an effect through prostaglandin hormone signaling, while MCT deliver dietary caloric energy (FO fatty acid contribution to dietary energy is largely negligible).

Felines were selected as test subjects as they have been assessed for response to intake of dietary lipids previously, but previous studies did not assess the global lipidome and did not compare the effects of FO and MCT alone and in combination. Cats can benefit from dietary lipids, but the degree to which they might respond to the intervention described here was not previously reported.

Relatively few studies have directly compared changes of diverse circulating lipid classes in response to dietary MCT and n-3 LCPUT in combination versus the provision of these oils alone. You et al. provided n-6 LCPUT and MCT alone and in combination via enteral infusions into the cannulated small intestine of rats and found that the fatty acid composition of the triglyceride fraction of plasma largely followed the differences in the n-6 LCPUT and MCT sources [45]. The effect of addition of FO to MCT on plasma NEFAs and the fatty acyl component of phospholipids has been assessed, although the identity of the phospholipid class (choline, inositol, ethanolamine, sphingolipid) was not analyzed and there was no control intervention that contained neither MCT nor FO [46]. The impact of either FO or MCT on lipoproteins and lipases in a patient with familial chylomicronemia was assessed, but NEFA, phospholipids, and other complex lipids were not reported and there was no combination of FO with MCT [47]. The effects of dietary coconut oil and FO on circulating NEFA, acylcarnitines, and lysophospholipids in cats [33] and dogs [48] has been reported. Compared to the current study, the Hall studies did not test intervention diets containing MCT without FO, the dietary levels of caprate (C:10) were 5-fold (feline study) to 20-fold (canine study) lower due to the use of coconut oil rather than purified MCT, there was no reported caprylate (C:8) in the foods, EPA (C20:5n3) was higher than DHA (C22:6n3), and DHA (C22:6n3) was present at levels an order of magnitude lower in the foods. Importantly, assessment of whether the effects were different between oils was not reported in felines.

Circulating levels of the ketone body BHBA are known to be elevated after administration of MCT [2]. The current data indicate that co-administration of FO+MCT resulted in an apparent increase in the production of BHBA beyond that produced by MCT alone. This observation has not been previously reported elsewhere. Enhanced conversion of MCFA to BHBA may at least partially explain why circulating levels of caprate (C10:0) in the FO+MCT diet group were less than half of the levels observed in the MCT diet group.

In many cases the effects of FO+MCT to alter lipid class levels was greater than for either oil alone, and simple mathematical addition of the effects of MCT to those of FO does not account for the increased effects of FO+MCT. It appears that when combined, FO+MCT acts synergistically. One of these metabolites that significantly increased in the FO+MCT group but was significantly decreased in the MCT group and relatively unaffected in the FO group was stearidonate (18:4n3), which has potential health benefits [49]. In contrast to the synergism observed for some lipid species, for the lysolipid subclasses of GPC, GPI, or GPE phospholipids, there was no differential effect of oil intervention on the number of significant changes from baseline, and their directionality was not different than it was in the class as a whole. Levels of biologically active NAAN were also altered by FO+MCT to an extent not observed for the FO or MCT oils interventions alone. Accumulation of N-acyl taurines has been implicated to increase insulin secretion, leading to β cell dysfunction in type 2 diabetes in mice [50]. Five of eight N-acyl taurines detected were significantly decreased in the FO+MCT group in this study, indicating that this combination may have beneficial effects on insulin regulation in cats. Pertinent to this observation, clinical chemistry analysis showed that blood glucose decreased in the FO+MCT group (from 84.6 to 78.4 mg/dL; *P* = 0.001) but was unchanged in the groups consuming either MCT or FO alone (see S4 Table). It might be expected that the FO+MCT combination would also produce physiological effects on satiety, neuromodulation, GI motility, and vascular tone. Future studies will be needed to assess the physiological consequences of combining these oils. In other cases, co-inclusion of FO in the FO+MCT food appears to have abrogated the effects of MCT alone. Arachidonic acid (C20:4n6) was only significantly changed (decreased) in the group that consumed the MCT food. This effect of MCT on arachidonic acid (C20:4n6) has previously been observed in a rat model [51].

Although structurally divergent, fatty acids in FO and MCT utilize common processes of beta oxidation, chain elongation, and (de)saturation for metabolism. Further, there is evidence for biochemical interaction of these dietary fats. Provision of MCT has previously been shown to increase the accumulation of long chain fatty acid-containing triglycerides in lymph fluid [45], both long chain triglycerides and MCT modulate fatty acid desaturase activity [52–55], MCT can participate as substrates for chain elongation prior to beta oxidation [56], and MCT have been shown to induce lipogenic fatty acid synthases [57]. These effects could potentially influence the composition of circulating lipids derived from dietary or endogenous sources but were not assessed in the current study. Additional studies are needed to determine the degree to which FO+MCT influenced activity of enzymes involved in fatty acid metabolism to produce the effects observed on circulating lipids.

Extensive effects of the FO+MCT food on circulating microbial postbiotics in the indole/indolic sulfate and phenol/phenolic sulfate classes in cats were observed in the current study. These classes of postbiotics are derived from the putrefaction of aromatic amino acids that originated from undigested protein having undergone microbial proteolysis in the large intestine [12]. With many of these metabolites considered to be uremic toxins that are correlated with the progression of renal failure, this combination of FO+MCT may provide a renal-protective effect [13]. To our knowledge, the observation that a combination of FO and MCT synergistically decrease circulating putrefactive postbiotics has not been previously published. The way in which FO+MCT produced a synergistic effect on circulating levels of microbiome postbiotics in this study is not readily apparent and additional research will need to be conducted to ascertain the mechanism. However, the documented physiologic effects of FO and MCT would be expected to impact gut microbiome composition, and indeed FO-derived PUFA as well as MCT have been shown to effect positive changes in the gut microbiome [14, 20] and to have anti-inflammatory effects and to modulate gut physiology [7, 16, 19, 23]. Gut barrier integrity is a factor that could influence the migration of microbial postbiotics into systemic circulation [58]. As one example of how FO+MCT might interact to influence microbial postbiotic levels, MCT can improve intestinal energy metabolism to increase gut barrier integrity [59, 60], while FO reduces inflammation that could otherwise decrease gut barrier integrity [61–63]. Although not assessed in the current study, it may be that the previously established effects of FO+MCT on microbiome composition, inflammatory state and gut barrier underpin the observed differences in circulating microbial postbiotic levels. In terms of the mechanism behind the differences observed specifically for the host-sulfated postbiotics, the decreases seen in sulfated forms of both indoles and phenols in the FO+MCT group may be due to a dearth of indole or phenol substrates available for host sulfation in the intestinal epithelia and liver. Alternatively, it may involve a direct effect of the FO+MCT food on the process of sulfation. However, since unconjugated indoles and phenols also decreased in tandem with their sulfated congeners, it is unlikely that the decrease in indoles and phenols is due to enhanced sulfation and urinary excretion, although this remains to be tested.

A novel aspect of this study stemmed from the use of caprylate (C8:0)-enriched MCT oil and DHA (C22:6n3)-enriched FO. Caprylate (C8:0) is known to exert strong metabolic effects relative to caprate (C10:0) and the transition fatty acids laurate (C12:0) and myristate (C14:0). DHA (C22:6n3) is known to produce stronger effects on neuromodulation than the more predominant FO fatty acid constituent EPA (C20:5n3). The extent of the effects of the FO+MCT combination are likely influenced by their proportions, as evidenced by comparison with a prior study in which cats were fed a control food; food with FO (0.23% EPA and 0.14% DPA); or food with FO (0.23% EPA and 0.14% DPA), and coconut oil (0.56% caprate [C10:0]) [33]. For example, little change was observed in the levels of laurate (12:0) in the FO+MCT group in this study, but an over 2-fold log increase was seen in the combination group in Hall et al, perhaps due to the use of coconut oil rather than MCT per se in that study. Opposite effects were seen in the two studies on levels of palmitoleate (16:1n7) and oleate (18:1), but levels of EPA (20:5n3), DPA (22:5n3), and DHA (22:6n3) were all increased relative to baseline. Of note, the control foods in these two studies differed in protein concentrations, and the studies were different lengths; this study used a 4-week time period, while Hall et al. was six months. Both of these factors could also have contributed to the differences seen.

The FO and MCT oils employed here are used as nutritional adjuvants in other species including humans and canine companion animals. It is to be expected that these results will translate to these and other species based on the published precedents cited herein and the overlapping metabolic processes these species in common. A limitation of this study could be the relatively short (2-week) washout feeding period prior to feeding the test foods, as more changes from baseline occurred in the control group than would be expected by chance (at alpha = 0.05). However, there was approximately a 2–4 times greater number of significant changes in the FO, MCT, and FO+MCT oil intervention groups than in the control group (see S4 Table). Additionally, knowing whether the effects observed in this study can extend into a longer term resulting in durable health benefits would be of interest. Nevertheless, strengths of this study are that the combination of FO+MCT could be assessed relative to each oil in isolation and that the use of paired data enabled each cat to serve as its own control to compare the changes from baseline rather than a post-interventional cross-sectional assessment.

In summary, the combination of two diverse oil types in cats led to effects on plasma metabolites that were not present in either oil alone, particularly in regard to microbiome metabolites.

## Supporting information

S1 Fig Random Forest analysis of predictors of group differences. CON, control; FO, fish oil; MCT, medium-chain fatty acid-containing triglycerides.
(TIF)

S1 Table As fed composition of the four food types used in the study. Bold font indicates differences among the foods.
(DOCX)

S2 Table Calculated composition of food used in the study. (DOCX)

S3 Table Characteristics of cats in the study. (DOCX)

S4 Table Plasma metabolite data. Cats were fed control food, food with MCT, food with FO, or food with MCT and FO. FO, fish oil; MCT, medium-chain fatty acid-containing triglycerides.
(XLSX)

S1 Checklist The ARRIVE guidelines checklist. (PDF)
