# Supplementary figures and images for: Docosahexaenoate-enriched fish oil and medium chain triglycerides shape the feline plasma lipidome and synergistically decrease circulating gut microbiome-derived putrefactive postbiotics
Source: PLoS One. 2020 Mar 12;15(3):e0229868. doi: 10.1371/journal.pone.0229868 (PMC7067441; doi:10.1371/journal.pone.0229868)

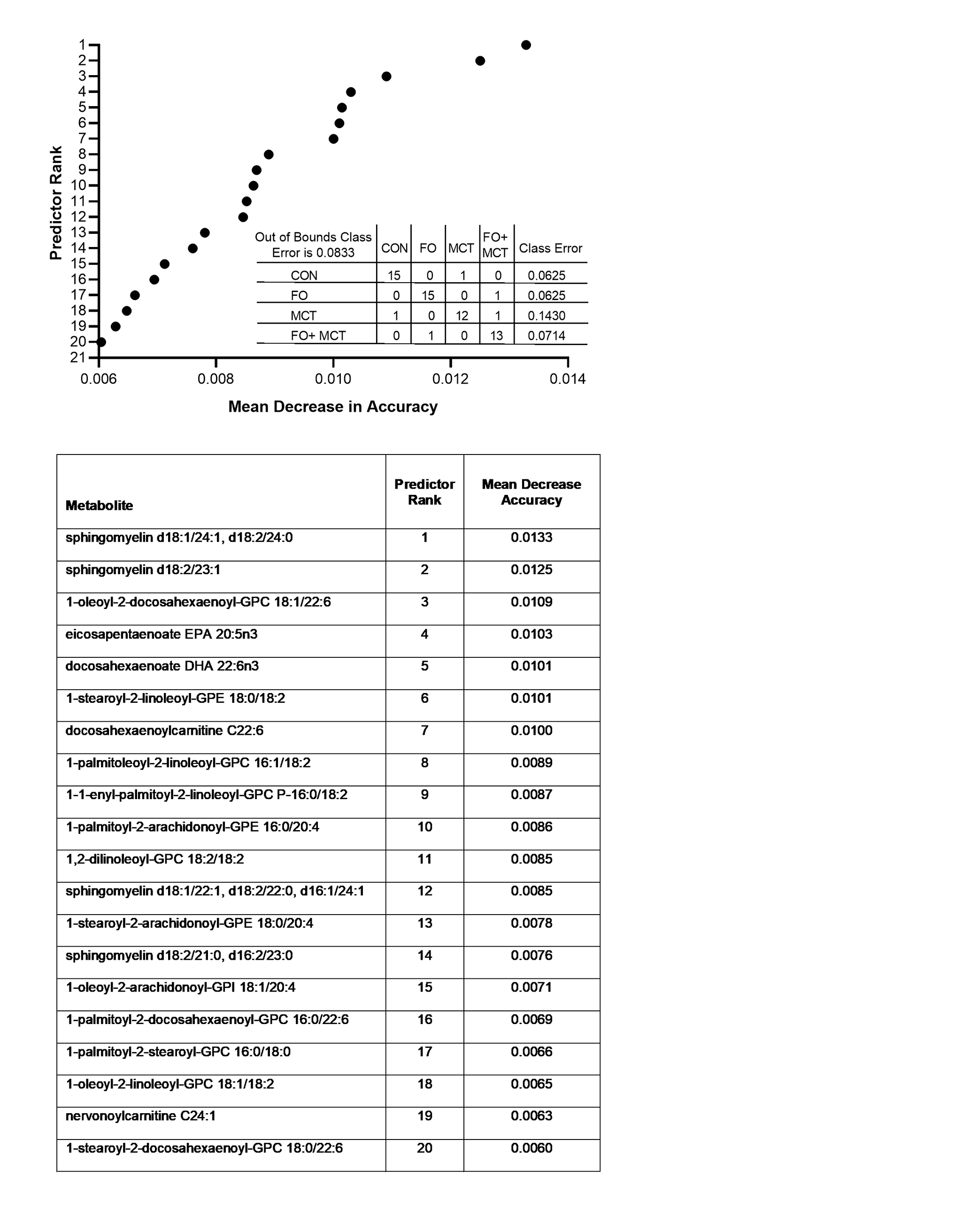

Supplement: S1 Fig — CON, control; FO, fish oil; MCT, medium-chain fatty acid-containing triglycerides. (TIF) [file pone.0229868.s001.tif]
