# Supplementary material for: Docosahexaenoate-enriched fish oil and medium chain triglycerides shape the feline plasma lipidome and synergistically decrease circulating gut microbiome-derived putrefactive postbiotics
Source: PLoS One. 2020 Mar 12;15(3):e0229868. doi: 10.1371/journal.pone.0229868 (PMC7067441; doi:10.1371/journal.pone.0229868)
Supplement: S1 Table — Bold font indicates differences among the foods. (DOCX) [file pone.0229868.s002.docx]

**S1 Table.** As fed composition of the four food types used in the study. Bold font indicates differences among the foods.

| **Ingredient, %** | **CON** | **MCT** | **FO** | **FO+MCT** |
| --- | --- | --- | --- | --- |
| Wet chicken | 25.64 | 25.64 | 25.64 | 25.64 |
| Corn gluten meal | 24.23 | 24.23 | 24.23 | 24.23 |
| Red wheat | 23.15 | 23.15 | 23.15 | 23.15 |
| Pork fat | 12.35 | 6.50 | 9.97 | 4.12 |
| Chicken meal | 2.09 | 2.09 | 2.09 | 2.09 |
| Beet pulp | 2.09 | 2.09 | 2.09 | 2.09 |
| Brown rice | 1.96 | 1.96 | 1.96 | 1.96 |
| Chicken liver | 1.67 | 1.67 | 1.67 | 1.67 |
| Palatant | 1.67 | 1.67 | 1.67 | 1.67 |
| Calcium sulfate | 1.44 | 1.44 | 1.44 | 1.44 |
| Lactic acid | 1.00 | 1.00 | 1.00 | 1.00 |
| Potassium chloride | 0.80 | 0.80 | 0.80 | 0.80 |
| Soybean oil | 0.42 | 0.42 | 0.42 | 0.42 |
| Sodium chloride, iodized | 0.38 | 0.38 | 0.38 | 0.38 |
| Choline chloride | 0.38 | 0.38 | 0.38 | 0.38 |
| Vitamin E | 0.36 | 0.36 | 0.36 | 0.36 |
| Taurine | 0.15 | 0.15 | 0.15 | 0.15 |
| Vitamin mix | 0.14 | 0.14 | 0.14 | 0.14 |
| Mineral mix | 0.05 | 0.05 | 0.05 | 0.05 |
| Fruit and vegetable blend | 0.03 | 0.03 | 0.03 | 0.03 |
| **Fish oil** | **0.00** | **0.00** | **2.38 (2.85*^a^*)** | **2.38 (2.85*^a^*)** |
| **Medium chain triglycerides** | **0.00** | **5.85 (7*^a^*)** | **0.00** | **5.85 (7*^a^*)** |
| ***^a^*** Dry matter basis. | | | | |

CON, control; FO, fish oil; MCT, medium-chain fatty acid-containing triglycerides.
