# Supplementary material for: Docosahexaenoate-enriched fish oil and medium chain triglycerides shape the feline plasma lipidome and synergistically decrease circulating gut microbiome-derived putrefactive postbiotics
Source: PLoS One. 2020 Mar 12;15(3):e0229868. doi: 10.1371/journal.pone.0229868 (PMC7067441; doi:10.1371/journal.pone.0229868)
Supplement: S2 Table — (DOCX) [file pone.0229868.s003.docx]

**S2 Table.** Calculated composition of food used in the study.

| **Food Component** | **Percent *^a^*** |
| --- | --- |
| Moisture | 7.5 |
| Protein | 35.4 |
| NFE | 33.7 |
| Fat | 22.0 |
| Total MCT*^b^* | 6.9 |
| Caproate (C6:0)*^b^* | 0.6 |
| Caprylate (C8:0)*^b^* | 3.6 |
| Caprate (C10:0)*^b^* | 2.7 |
| Total EPA + DHA*^c^* | 1.1 |
| EPA (C20:5n3)*^c^* | 0.14 |
| DHA (C22:6n3)*^c^* | 1.0 |
| Crude Fiber | 1.5 |
| Dietary Fiber | 6.8 |
| Soluble Fiber | 1.4 |
| Insoluble Fiber | 5.7 |
| Ash | 7.4 |

***^a^*** All on a dry matter basis except for moisture. *^b^* Only in the MCT and FO+MCT foods. *^c^* Only in the FO and FO+MCT foods.

DHA, docosahexaenoate; EPA, eicosapentaenoate; FO, fish oil; MCT, medium-chain fatty acid-containing triglycerides; NFE, nitrogen-free extract.
