# Supplementary material for: Docosahexaenoate-enriched fish oil and medium chain triglycerides shape the feline plasma lipidome and synergistically decrease circulating gut microbiome-derived putrefactive postbiotics
Source: PLoS One. 2020 Mar 12;15(3):e0229868. doi: 10.1371/journal.pone.0229868 (PMC7067441; doi:10.1371/journal.pone.0229868)
Supplement: S3 Table — (DOCX) [file pone.0229868.s004.docx]

**S3 Table.** Characteristics of cats in the study.

| **Subject** | **Diet Type** | **Age, y** | **Weight, kg** | **Sex** | **Health Issue** | **Housing** | **Complete Collections** | **Completed Trial** |
| --- | --- | --- | --- | --- | --- | --- | --- | --- |
| 1 | Feline Adult | 5.6 | 4.3 | Spayed Female | None | Single | Y | Y |
| 2 | Feline Adult + Fish Oil | 3.4 | 5.6 | Spayed Female | None | Single | Y | Y |
| 3 | Feline Adult + MCT | 3.6 | 6.1 | Neutered Male | None | Group | Y | Y |
| 4 | Feline Adult + Fish Oil | 3.7 | 4.2 | Spayed Female | None | Group | Y | Y |
| 5 | Feline Adult + MCT | 7.9 | 6.3 | Neutered Male | None | Group | Y | Y |
| 6 | Feline Adult + Fish Oil | 3.4 | 4.6 | Spayed Female | None | Group | Y | Y |
| 7 | Feline Adult + Fish Oil | 7.9 | 4.3 | Spayed Female | None | Single | Y | Y |
| 8 | Feline Adult | 3.7 | 3.4 | Spayed Female | None | Group | Y | Y |
| 9 | Feline Adult + Fish Oil | 7.9 | 5.9 | Spayed Female | None | Group | Y | Y |
| 10 | Feline Adult + Fish Oil & MCT | 7.9 | 5.2 | Neutered Male | None | Group | Y | Y |
| 11 | Feline Adult | 3.4 | 7.2 | Neutered Male | None | Group | Y | Y |
| 12 | Feline Adult + MCT | 7.9 | 5.3 | Spayed Female | None | Group | Y | Y |
| 13 | Feline Adult + Fish Oil & MCT | 5.5 | 5.3 | Neutered Male | None | Single | Y | Y |
| 14 | Feline Adult + Fish Oil | 5.5 | 3.9 | Spayed Female | None | Single | Y | Y |
| 15 | Feline Adult + Fish Oil | 3.6 | 5.6 | Neutered Male | None | Single | Y | Y |
| 16 | Feline Adult + MCT | 7.9 | 5.1 | Neutered Male | None | Group | Y | Y |
| 17 | Feline Adult | 3.5 | 6.7 | Neutered Male | None | Single | Y | Y |
| 18 | Feline Adult + MCT | 4.9 | 4.2 | Spayed Female | None | Single | N | Y |
| 19 | Feline Adult | 3.4 | 4.5 | Spayed Female | None | Group | Y | Y |
| 20 | Feline Adult | 2 | 5.3 | Spayed Female | None | Single | Y | Y |
| 21 | Feline Adult | 7.9 | 6.0 | Spayed Female | None | Single | Y | Y |
| 22 | Feline Adult | 8.2 | 5.4 | Neutered Male | None | Group | Y | Y |
| 23 | Feline Adult | 16.6 | 3.3 | Spayed Female | None | Group | Y | Y |
| 24 | Feline Adult + Fish Oil & MCT | 7.9 | 5.3 | Spayed Female | None | Group | Y | Y |
| 25 | Feline Adult + MCT | 3.7 | 5.8 | Neutered Male | None | Single | Y | Y |
| 26 | Feline Adult + Fish Oil & MCT | 3.7 | 6.3 | Neutered Male | None | Group | Y | Y |
| 27 | Feline Adult | 3.6 | 5.5 | Spayed Female | None | Group | Y | Y |
| 28 | Feline Adult + MCT | 7.9 | 5.5 | Spayed Female | None | Group | Y | Y |
| 29 | Feline Adult + Fish Oil | 3.5 | 6.0 | Neutered Male | None | Group | Y | Y |
| 30 | Feline Adult + Fish Oil & MCT | 6.2 | 4.4 | Spayed Female | None | Single | Y | Y |
| 31 | Feline Adult + MCT | 7 | 4.4 | Spayed Female | None | Single | Y | Y |
| 32 | Feline Adult + MCT | 6.2 | 4.2 | Spayed Female | None | Single | Y | Y |
| 33 | Feline Adult + Fish Oil & MCT | 3.6 | 6.3 | Spayed Female | None | Single | Y | Y |
| 34 | Feline Adult + Fish Oil | 3.6 | 5.2 | Spayed Female | None | Group | Y | Y |
| 35 | Feline Adult + MCT | 3.5 | 3.9 | Spayed Female | None | Group | Y | Y |
| 36 | Feline Adult + Fish Oil & MCT | 3.5 | 4.2 | Spayed Female | None | Group | Y | Y |
| 37 | Feline Adult + Fish Oil | 7 | 4.1 | Spayed Female | None | Group | Y | Y |
| 38 | Feline Adult + Fish Oil | 7.9 | 5.2 | Spayed Female | None | Group | Y | Y |
| 39 | Feline Adult + MCT | 3.7 | 5.6 | Spayed Female | None | Group | Y | Y |
| 40 | Feline Adult | 7.9 | 4.9 | Spayed Female | None | Group | Y | Y |
| 41 | Feline Adult + Fish Oil & MCT | 6.6 | 3.0 | Spayed Female | None | Group | Y | Y |
| 42 | Feline Adult + Fish Oil & MCT | 3.7 | 6.4 | Neutered Male | None | Group | Y | Y |
| 43 | Feline Adult + Fish Oil | 7.9 | 6.9 | Neutered Male | None | Group | Y | Y |
| 44 | Feline Adult + MCT | 6.2 | 6.8 | Neutered Male | None | Single | Y | Y |
| 45 | Feline Adult | 7.9 | 4.2 | Spayed Female | None | Group | Y | Y |
| 46 | Feline Adult + MCT | 6.6 | 3.9 | Spayed Female | None | Group | Y | Y |
| 47 | Feline Adult + Fish Oil & MCT | 6.2 | 4.8 | Spayed Female | None | Group | N | N |
| 48 | Feline Adult + Fish Oil | 6.4 | 5.8 | Neutered Male | None | Single | Y | Y |
| 49 | Feline Adult + Fish Oil & MCT | 3.6 | 4.5 | Spayed Female | None | Group | Y | Y |
| 50 | Feline Adult | 6.2 | 7.0 | Neutered Male | None | Group | Y | Y |
| 51 | Feline Adult + Fish Oil & MCT | 3.7 | 6.1 | Neutered Male | None | Single | Y | Y |
| 52 | Feline Adult | 7.8 | 5.3 | Neutered Male | None | Single | Y | Y |
| 53 | Feline Adult + Fish Oil & MCT | 7.9 | 5.6 | Neutered Male | None | Group | Y | Y |
| 54 | Feline Adult + MCT | 3.6 | 5.2 | Spayed Female | None | Single | Y | Y |
| 55 | Feline Adult + Fish Oil & MCT | 7.9 | 5.4 | Spayed Female | None | Group | N | Y |
| 56 | Feline Adult + Fish Oil | 5.7 | 5.1 | Spayed Female | Hip Dysplasia, Hypertension | Single | Y | Y |
| 57 | Feline Adult + Fish Oil | 5.6 | 6.0 | Neutered Male | None | Group | Y | Y |
| 58 | Feline Adult | 6.5 | 6.9 | Neutered Male | None | Group | Y | Y |
| 59 | Feline Adult | 5.5 | 4.2 | Spayed Female | None | Single | Y | Y |
| 60 | Feline Adult + MCT | 5.4 | 6.4 | Neutered Male | None | Group | Y | Y |
| 61 | Feline Adult + MCT | 3.6 | 4.6 | Spayed Female | None | Group | N | Y |
| 62 | Feline Adult + Fish Oil & MCT | 6.2 | 5.5 | Spayed Female | None | Single | Y | Y |
| 63 | Feline Adult + Fish Oil | 7.1 | 4.7 | Neutered Male | None | Group | Y | Y |
| 64 | Feline Adult + Fish Oil & MCT | 3.9 | 5.9 | Spayed Female | None | Single | Y | Y |
